# Oviductal extracellular vesicles miRNA cargo varies in response to embryos and their quality

**DOI:** 10.1186/s12864-024-10429-5

**Published:** 2024-05-27

**Authors:** Meriem Hamdi, José María Sánchez, Beatriz Fernandez-Fuertes, Diogo Ribeiro Câmara, Heinrich Bollwein, Dimitrios Rizos, Stefan Bauersachs, Carmen Almiñana

**Affiliations:** 1 Institute of Veterinary Anatomy, Vetsuisse Faculty Zurich, University of Zurich, Lindau, ZH 8315 Switzerland; 2 Department of Animal Reproduction, Instituto Nacional de Investigación y Tecnología Agraria y Alimentaria (INIA-CSIC), Madrid, Spain; 3 Department of Veterinary Medicine, Federal University of Alagoas, Viçosa, AL Brazil; 4 Clinic of Reproductive Medicine, Vetsuisse Faculty, University of Zurich, Lindau, ZH 8315 Switzerland; 5 Department of Reproductive Endocrinology, University Hospital Zurich, Zurich, Switzerland

**Keywords:** Oviduct, Extracellular vesicles, Exosomes, Oviductal epithelial cells, Embryo, Bos taurus, RNA-sequencing, miRNA

## Abstract

**Background:**

Increasing evidence points to an active role of oviductal extracellular vesicles (oEVs) in the early embryo-maternal dialogue. However, it remains unclear whether oEVs contribute to the recognition of the presence of embryos and their quality in the oviduct. Hence, we examined whether the molecular cargo of oEVs secreted by bovine oviduct epithelial cells (BOEC) differs depending on the presence of good (≥ 8 cells, G) or poor (< 8 cells, P) quality embryos. In addition, differences in RNA profiles between G and P embryos were analyzed in attempt to distinguish oEVs and embryonic EVs cargos.

**Methods:**

For this purpose, primary BOEC were co-cultured with in vitro produced embryos (IVP) 53 h post fertilization as follows: BOEC with G embryos (BGE); BOEC with P embryos (BPE); G embryos alone (GE); P embryos alone (PE); BOEC alone (B) and medium control (M). After 24 h of co-culture, conditioned media were collected from all groups and EVs were isolated and characterized. MicroRNA profiling of EVs and embryos was performed by small RNA-sequencing.

**Results:**

In EVs, 84 miRNAs were identified, with 8 differentially abundant (DA) miRNAs for BGE vs. B and 4 for BPE vs. B (*P*-value < 0.01). In embryos, 187 miRNAs were identified, with 12 DA miRNAs for BGE vs. BPE, 3 for G vs. P, 8 for BGE vs. GE, and 11 for BPE vs. PE (*P*-value < 0.01).

**Conclusions:**

These results indicated that oEVs are involved in the oviductal-embryo recognition and pointed to specific miRNAs with signaling and supporting roles during early embryo development.

**Supplementary Information:**

The online version contains supplementary material available at 10.1186/s12864-024-10429-5.

## Background

Assisted reproductive technologies (ARTs) have come a long way in the last 30 years both in animals and humans, allowing in vitro embryo development until the blastocyst stage and successful pregnancy after embryo transfer [1]. In livestock, a substantial increase in the number of in vitro produced (IVP) embryos has been registered during the last years. In cattle for example, the International Embryo Technology Society (IETS) registered the production of more than 1.15 million IVP embryos and the transfer of 878,181 IVP embryos worldwide in 2020, 12% and 10% more, respectively, than in 2019. Although the numbers show that cattle IVP is continuously increasing, the efficiency of current IVP systems is far from optimal. Only 30% of cattle receiving IVP embryos will deliver a live calf, with 60% of these pregnancies failing during the first 6 weeks of gestation [2]. The persistent struggle is to maintain the pregnancy rate close to 50%, especially with IVP or cryopreserved embryos [1, 2].

This low efficiency has been related to a gradual decrease in fertility observed in cattle herds [3], but also to the suboptimal quality of IVP compared to in vivo derived embryos [4]. The lack of maternal signals during all steps of IVP has been postulated as the main reason for the lower embryo quality [5]. Indeed, the environment in which the early embryo develops has a critical influence on its growth and fate not only in the short- but also in the long-term [6]. Fetal and placental developmental defects such as the large-offspring syndrome have been associated to embryo IVP techniques in cattle and in other species [7–9].

Increasing evidence is pointing to the role of extracellular vesicles (EVs) in early reproductive events and promoting successful pregnancy [10–12]. EVs are nano cargo-bearing vesicles secreted by cells and play a central role in cell-to-cell communication [13]. EVs are present in reproductive fluids (follicular, oviductal and uterine fluids) among other biofluids [14]. They carry different biomolecules such as proteins, nucleic acids (different types of RNAs and DNA), lipids, and metabolites that provide a snapshot of the parental cells at the time of secretion but also transfer specific signaling and regulatory molecules [15]. Given these intrinsic features, EVs have been pointed as biomarkers for diagnosis and prognosis of cancer and other diseases in recent years [16, 17].

For the oviduct, an increasing number of studies are evidencing the role of oviductal EVs (oEVs) as active players in the early embryo-maternal dialogue and supporting early embryo development [18–21]. So far, it has been shown that oEVs interact with both spermatozoa and oocytes, while exerting a functional effect [22–25]. They also have an impact during fertilization, for example, by reducing polyspermy [26]. Oviductal EVs can also be taken up by embryos at different developmental stages [27, 28], boosting their development, cryotolerance [27, 29, 30] and regulating their transcriptome [30, 31]. To understand their functional effects, the molecular cargo of oEVs from cyclic and pregnant animals has been examined, pointing to a variety of cargo: from proteins, mRNA, miRNAs to metabolites as potential molecules supporting gametes/embryo-oviductal interactions and embryo development [32–34]. However, whether oEVs are a part of the recognition system of embryo quality in the oviduct remains to be explored.

Considering that the oviduct can distinguish between the presence of spermatozoa and oocytes [35], and even between subtle differences in X- and Y-bearing spermatozoa [36], it is likely that it can also distinguish between good or poor quality embryos. To the best of our knowledge, this has not been explored yet in the oviduct, in contrast to the endometrium. The endometrium has been proven to be a sensor of embryo quality, preventing inappropriate investment in poorly viable embryos [37]. In cattle, transcriptomic signatures were identified dependent on the embryo origin (i.e., embryos derived from artificial insemination (AI), in vitro fertilization (IVF) or somatic cell nuclear transfer (SCNT)) [38, 39]. Brosens et al. [40] showed that when conditioned medium from developmentally competent human embryos was injected into the uterine horns of mice, this triggered a very specific transcriptional response in the uterus, activating gene networks enriched in metabolic enzymes and implantation factors. In contrast, conditioned culture medium from low-quality human embryos exerted a stress response in the murine uterus [40]. In the oviduct, one recent study has pointed to the oviductal recognition of EVs only from good embryos by transcriptomic alteration of the oviduct, but not from degenerated embryos [41].

Here, we hypothesized that oEVs play an active role in the embryo recognition system in the oviduct. Oviductal epithelial cells can distinguish between embryos of good (G) and poor (P) quality and in response, secrete oEVs with different cargo. Therefore, the aim of this study was to determine differences in oEVs cargo in the presence of G or P embryos, and in their absence. Since EVs in the presence of embryos are a mixture of EVs of embryonic and oviductal origin, EVs derived from embryos cultured alone were also examined. In addition, differences in RNA profiles between G and P embryos were analyzed in attempt to distinguish oEVs and embryonic EVs cargos. For this purpose, an in vitro model based on the co-culture of primary bovine oviductal epithelial cells (BOEC) with IVP embryos was used. MicroRNA profiling of culture media EVs as well as G and P embryos was performed. This integrative approach allowed to determine whether the molecular cargo of oEVs secreted by BOEC differs depending on the embryo presence and its quality, while pinpointing specific miRNAs contributing to embryo recognition signaling and support of embryo development.

## Methods

All the chemicals were purchased from Merck & Cie (9470 Buchs, Switzerland), unless otherwise stated.

### Experimental design

**Fig. 1:**
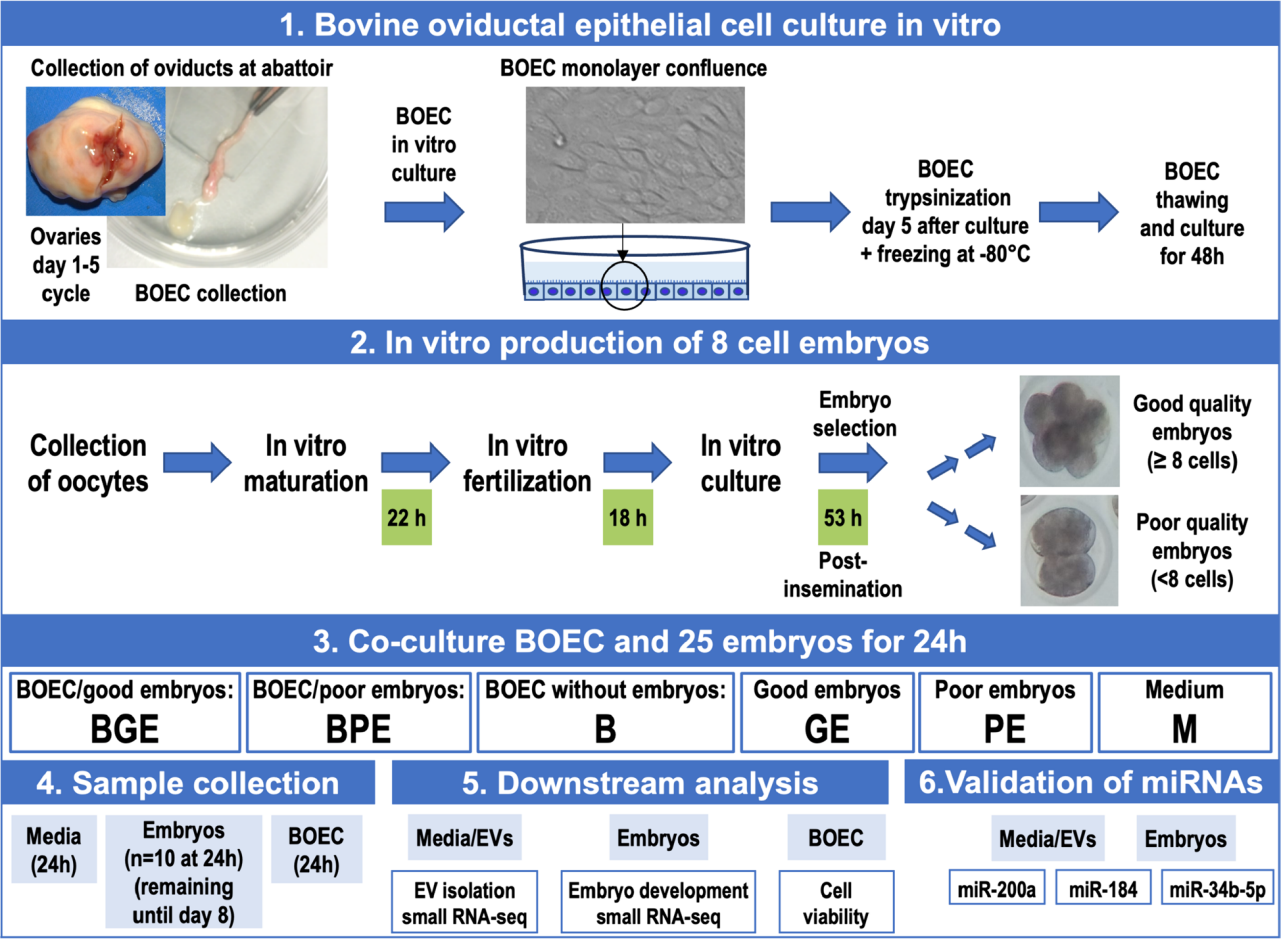
Schematic representation of experimental design of the study

To characterize and examine the miRNA cargo of EVs derived from the co-culture of BOEC with early embryos of G and P quality, primary culture of BOEC and in vitro produced bovine embryos were conducted. A schematic diagram of the experimental design is shown in Fig. 1. Presumptive zygotes were cultured until 53 h after fertilization, and then classified according to their developmental stage and quality based on their morphological aspect. Embryos with 8 or more cells and good morphology aspect (similar sized cells, similar thickness of zona pellucida) were categorized as good quality (G). Embryos with less than 8 cells (but cleaved) and/or not good morphology (but not fragmented) were classified as poor-quality embryos (P). Embryos which at 53 h were not developed (not cleaved) or were degenerated or fragmented were not used. The classification of the embryos in good- and poor-quality was based on previous studies, selecting a pool of fast, moderate and slow embryos in the “good embryos”, since selecting fast embryos only could bias the selection towards more male embryos [42–45]. Then, both G and P embryos were co-cultured in groups of 25 embryos with frozen-thawed BOEC monolayers (~ 85% of confluence) or alone for 24 h, as follows: 1) BGE: BOEC co-cultured with G embryos; BPE: BOEC co-cultured with P embryos; GE: culture of G embryos without cells; PE: culture of P embryos without cells; and as controls, B: BOEC alone as well as M: medium alone. After 24 h of co-culture, conditioned media, embryos and BOEC were collected from all groups. Besides, a group of embryos from each experimental group was further cultured in vitro without BOEC until day 8 to evaluate embryo development. Subsequently, EVs were isolated from all groups and characterized. MicroRNA profiling of oEVs and embryos from all experimental groups was performed by low-input small RNA-sequencing. A total of 12 replicates were conducted. Conditioned media from 2 replicates of each experimental group were pooled to increase EVs yield, providing a total of 4 replicates for each EVs experimental group and controls (8 replicates): EV_BGE_R1-R4; EV_BPE_R1-R4; EV_B_R1-R4; EV_GE_R1-R4; EV_PE_R1-R4; and M_R1-R4. The remaining 4 replicates were used for EVs characterization experiments. For embryos, 10 embryos of each experimental group were pooled for one replicate and a total of 4 replicates were used for each embryo experimental group: EB_BGE_R1-R4; EB_BPE_R1-R4; EB_GE_R1-R4; and EB_PE_R1-R4. For BOEC, a total of 3 replicates per each experimental group were used for cell viability assay.

### Collection of bovine oviducts and ovaries and in vitro culture of BOEC

Bovine oviducts were collected from cyclic heifers at a local abattoir (Transformación Ganadera de Leganés SA; in Madrid, Spain) and transported on ice to the laboratory within 2–3 h. Only oviducts ipsilateral to ovaries at early ovulatory stages (from Day 1 to 5 of the estrous cycle) were used to isolate BOEC as previously described [46]. Each oviduct was trimmed free of tissue and then, the oviductal mucosa was collected by squeezing and washed twice with PBS. Subsequently, the collected cells were centrifuged at 300 g for 7 min. To minimize animal variability, the oviductal mucosa from 10 oviducts were pooled and the final pellet was suspended in 1 ml of prewarmed culture medium TCM199 (M4530-1L, Sigma) supplemented with 10% of fetal calf serum (FCS, F2442-50ML, Sigma) and 1X of antibiotic–antimycotic (A5955-20ML, Sigma), then passed 10 times through a 25 G syringe needle to obtain a single cells suspension. Final concentration was adjusted to 2 × 10^6^ cells/ml and cultured in 35 mm petri dishes, at 38.5 °C, 5% CO_2_ and saturated humidity. Forty-eight hours after seeding, the culture media were completely renewed, then half of it was replaced every 48 h. At day 5, when cells reached 80–90% of confluence, they were frozen following the protocol previously described [47]. Briefly, monolayers of cells were treated with 1 ml of PBS supplemented with Trypsin–EDTA (0.5%) (GibcoTM15400054) for 7 to 9 min, to detach them from the culture plate. Then, the enzyme was inactivated by adding 2 ml of pre-warmed TCM199 supplemented with 10% of FCS. The obtained mixture was centrifuged at 300 g during 5 min, and the resulted cell pellet was diluted in 1.5 ml of FCS supplemented with 10% of Dimethyl sulfoxide (DMSO, D4540, Sigma) and frozen at –80 °C.

### In vitro production of embryos

Immature cumulus oocyte complexes (COCs) were aspirated from 2–8 mm follicles, from ovaries collected at the local abattoir. Then, COCs were in vitro matured and fertilized, as previously described [48]. For all experiments, in vitro fertilization was performed with conventional semen, by using frozen/thawed semen from a pool of two bulls from the same ejaculate, with high in vivo fertility (non-return rate) and previously tested for IVF (kindly donated by Spanish Association of Breeders of Selected Cattle of the Asturian Valley Breed (ASEAVA)). Presumptive zygotes (18–22 h after insemination) were completely denuded of cumulus cells by vortexing during 3 min. Then, groups of 25 putative zygotes were cultured in 500 µL of Synthetic Oviductal Fluid with amino acids (SOFaa) supplemented with 5% EV-depleted fetal calf serum (d-FCS), obtained after ultracentrifugation for 18 h, as previously described [49]. The culture of zygotes was performed in four-well dishes under 5% CO_2_, 5% O_2_ and 90% N_2_ at 38.5 °C. At 53 h post-insemination (normal embryos should be at the 8 cells stage), embryos were classified based on their morphology as G (≥ 8 cells) and *P* (< 8 cells) quality embryos. Embryos used for blastocyst rate assessment were cultured until day 8 of development in SOFaa under 5% CO_2_, 5% O_2_ and 90% N_2_ at 38.5 °C.

### Co-culture of BOEC with early embryos

Forty-eight hours before co-culture of BOEC with embryos, BOEC were thawed with prewarmed TCM199 and centrifuged at 300 g for 5 min to remove remaining DMSO. The cells were cultured with TCM 199 supplemented with EV-depleted FCS in 4-well petri dishes at 38.5 °C and 5% CO_2_ until reaching 80% of confluence.

On the day of the co-culture, embryos classified as G or P quality were randomly allocated in groups of 25 embryos with or without BOEC according to the experimental design (BGE, BPE, GE, and PE). Additionally, controls of BOEC (B) and media (M) without BOEC nor embryos were cultured/incubated alone and treated in the same manner as the experimental groups. No FCS or BSA was used during co-culture.

### Collection of conditioned media and embryos after BOEC-embryo co-culture

Twenty-four hours after co-culture, 500 µL of the conditioned media were collected from each experimental group. Conditioned media were centrifuged at 300 g for 15 min, followed by 2,000 g for 15 min to remove cell debris. Media were frozen and stored at –80 °C until EV isolation and downstream analysis. Ten embryos per experimental group were also snap-frozen and stored at –80 °C until use for small RNA-seq. The remaining embryos were maintained in culture until blastocyst stage (day 8 after fertilization) to assess their embryo development rate and to confirm the appropriateness of the embryo quality selection. Moreover, BOEC from 4 replicates of each experimental group were used to evaluate cell viability. All frozen samples (conditioned media, embryos and BOEC) were transported in dry ice to the laboratories of the University of Zurich for further processing.

### Embryo development and cell viability assessment

Blastocyst rates were evaluated at day 7 and 8 of embryo culture and defined as the total number of blastocysts on day 7 or 8 divided by the total number of oocytes selected for maturation that were fertilized. The viability of BOEC after the co-culture was assessed by double staining with Propidium Iodide (PI, P4170-25MG, Sigma) and Hoechst 33342 (B2261-25MG, Sigma) and observed under fluorescent microscope (Nikon Eclipse TE 300) [27].

### Isolation of EVs from conditioned media

The conditioned media from all experimental groups (including M control group) were thawed on ice, centrifuged at 12,000 g for 30 min at 4 °C to remove large vesicles and other debris. Subsequently, EVs were isolated using size exclusion chromatography (SEC) columns (PURE-EV®, Hansa BioMed, ANAWA/Biotrend, Kloten, Switzerland) followed by ultracentrifugation, as previously described [46]. Briefly, after discarding the buffer from the SEC columns, they were washed with 30 mL of PBS and subsequently loaded with the samples. When all the sample was completely within the column, 11 mL of PBS were loaded to avoid the column drying. After discarding the first 3 mL (void volume of the column), the following 2 ml fraction, which contains the EVs, was collected. This fraction was ultracentrifuged at 100,000 g for 90 min at 4 °C to precipitate the EVs by using a swing-out Beckman rotor (TLS-55), Beckman tubes (No. 344057), and Beckman Optima MAX-XP ultracentrifuge (Beckman Coulter International S.A.). The final EV pellet was resuspended in 20 µL of PBS supplemented with 25 mM of trehalose (TRE, T0167, Sigma), as described in Almiñana et al. [50], and stored at –80 °C for EV characterization, RNA isolation, and small RNA-sequencing.

### Characterization of EVs

Extracellular vesicles preparations from all experimental groups were analyzed by transmission electron microscopy (TEM) as previously described [51]. Each sample for TEM analysis represented a pool of three different replicates of EVs samples from the same experimental group (6 pools: BGE, BPE, BE, GE, B, and M).

Nanoparticle tracking analysis (NTA) was carried out on a NanoSight NS300 (Malvern Panalytical, Westborough, MA, USA) equipped with laser 45 mW at 488 nm and an automated syringe sampler. To ensure an appropriate measurement, EV samples were diluted 1:20 in PBS to detect around 20 and 100 particles per frame. PBS was also measured as a negative control. Samples were loaded into 1 ml syringes and injected in continuous flow with a syringe pump speed of 50 µl/s at 24.6 to 24.7 °C. Each sample was measured in quintuplicate with the same camera settings level of 11 and an acquisition time of 30 s. After capture, the videos were analyzed using NanoSight Software NTA 3.1 Build 3.1.46 with a detection threshold set up at 2. Autofocus was adjusted so that indistinct particles were avoided. Two replicates of each experimental group were analyzed by NTA and measurements of mean particle size, mode, and concentration (particles/ml) were performed.

Flow cytometry analysis was performed using CytoFlex Flow Cytometer (Beckman Coulter, Fullerton, CA, USA) equipped with a violet (405 nm, 80 mW), blue (488 nm, 50 mW) and red (638 nm, 50 mW) laser. To resolve nanoparticles from noise, the CytoFLEX was configured to a violet laser detector to collect side scatter (VSSC). The VSSC threshold was set below the 100 nm bead population. For detecting fluorescein isothiocyanate (FITC) and phycoerythrin (PE) fluorescence, the 525/40 nm and 585/42 nm bandpass filters were used, respectively. Presence of nanosized objects in samples was controlled and confirmed by nanobeads (Spherotech NFPPS-52-4 K Nano Fluorescent Size Standard Kit) (Supplementary Figure S1). The flow rate was set at 30 µl/min. To prevent cross contamination between samples, PBS solution was run through the fluidics system of the cytometer for 5 s between samples. Briefly, EVs samples were prepared by incubating 10 µl of EVs sample from each experimental group with 30 µl of the mixture of the following antibodies: primary FITC-conjugated anti-human CD63 antibody (Abcam 18235); and primary RPE-conjugated anti-human CD9 antibody (Bio-Rad MCA469PE). Then, the mixture of EVs and antibodies was diluted to a final volume of 100 µl with PBS and incubated for 30 min at RT with continuous shaking. The flow cytometric analysis was restricted to the EVs based on their characteristic properties in the forward scatter (FSC) and violet side scatter (SSC). A gate was established to detect EVs based on their size (nanoparticles with a diameter between 100 to 300 nm) to distinguish true events from electronic noise and increase the specificity of EVs detection events in the EV gate. As negative control, PBS plus antibodies was used (Supplementary Figure S1). Data analysis was performed with CytExpert 2.4.0.28 Software (Beckman Coulter, Fullerton, CA, USA).

Western blotting was performed for known EV markers (membrane: CD9 and cytosolic: HSP70 and ANXA2). Only in vitro oEVs (pool samples) were used for Western blot experiments due to the limitations in the protein concentration of the samples, particularly from embryonic EVs. In vitro large vesicles of BOEC conditioned media (large EVs) obtained after centrifugation at 12,000 g were also used in Western blot experiments, in order to show that EV experimental samples contained mostly small EV population positive for the known EV markers in contrast to large vesicles [50–52]. Western blotting was performed as previously described [51]. Antibodies and their dilutions used for Western blotting experiments were as follows: primary antibodies: Anti-CD9 Mouse Monoclonal Antibody (Clone MM2/57, MCA469GT, Bio-Rad), 1:500; Anti-HSP70 Mouse Monoclonal Antibody (Santa Cruz sc-66048), 1:500; Anti-ANXA2 Mouse Monoclonal Antibody (Santa Cruz sc-28385), 1:500; secondary antibody: Anti-mouse m-IgGκ BP-HRP (Santa Cruz sc-516102), 1:10000.

Characterization experiments were performed with 2 replicates, since there were no more samples left, particularly from the embryonic EVs groups.

### MicroRNA profiling of EVs and embryos by low input small RNA-sequencing

#### RNA isolation, quantification and assessment of RNA profiles

To isolate RNA from EVs and embryo samples, QIAzol lysis reagent (QIAGEN AG, Hombrechtikon, Switzerland) followed by miRNeasy micro kit (QIAGEN) was used according to the manufacturer´s instructions. Then, RNA concentration was measured by Agilent High Sensitivity RNA ScreenTape® (Agilent TapeStation, Agilent Technologies Schweiz AG, Basel, Switzerland) for RNA quantity and to obtain electrophoresis profiles of EVs and embryo RNA samples. The EV samples and embryo samples with the highest RNA concentration and best quality were selected for preparation of small RNA-seq libraries (RNA Integrity Number (RIN) range for embryo samples: 6.3–7.6; RNA concentration embryos: 350–940 pg/µl and EVs: 50–140 pg/µl). In total, 34 libraries were prepared: 18 libraries for EVs with 3 replicates/EV experimental group (6 groups) and 16 libraries for embryos: 4 replicates/experimental group (4 groups).

#### RNA library preparation and sequencing

RNA-Seq library preparation and sequencing were performed at the Functional Genomics Center Zurich (FGCZ; https://fgcz.ch/). Library preparation started from approximately 0.5 ng of total RNA for EVs and 1 ng for embryos by using RealSeq®-AC miRNA Library Kit for Illumina® sequencing (cat. no. 500–00048, BioCat GmbH, Heidelberg, Germany) following manufacturer´s instructions. Sequencing of the obtained libraries was conducted on an Illumina NovaSeq 6000 instrument. Pooled barcoded libraries were run on one SP flow cell with 100 bp single-reads.

### RNA-seq data analysis

Fastq files were processed using Trimmomatic (Galaxy version 0.38.1) to remove the first base from the start of the read, remove bases from the beginning of the read with a phred quality score threshold of 30, cut the read to 40 bases, perform sliding window trimming (average across 5 bases, average quality = 30). In addition, adaptor sequences (TGGAATTCTCGGGTGCCAAGG) were trimmed, low quality ends removed (phred quality score threshold of 30) and reads shorter than 15 bases discarded with Trim Galore! Quality and adapter trimmer (Galaxy Version 0.6.3). Collapse sequences (Galaxy Version 1.0.1) was used to get unique sequences and corresponding counts for each sample. The sequence and count information of each sample was joined to generate a read count table with the unique sequences as identifier column. To remove sequences with neglectable counts, filtering was performed with Filter counttable by CPM cutoff (Galaxy Version 1.2) (CPM cutoff 2.0, sample cutoff 4). This resulted in a total of 48,833 sequences which were compared with NCBI BLAST + blastn (Galaxy Version 2.10.1 + galaxy1) (blastn-short) with a variety of non-coding and coding sequence collections including bovine, porcine, equine, murine, and human miRNA sequences (mature and stem-loop sequences, miRBase version 22.1), RFAM 14.7 sequences, bovine tRNA sequences, bovine and human NCBI Refseq RNA sequences, and bovine piRNAs derived from piRBase release 2.0 (http://bigdata.ibp.ac.cn/piRBase). Read counts for miRNAs were summarized for isomiR sequences belonging to the same mature miRNA. Differential expression analysis was performed using the Bioconductor package EdgeR (https://bioconductor.org/packages/edgeR/) [53].

To confirm MiRDeep2 results in consideration of miRNA annotation complications and limitations [54], an additional sequence data analysis was performed essentially as previously described [55]. Collapse sequences (Galaxy Version 1.0.1) was used to get unique sequences and corresponding counts for each sample. The sequence and count information of each sample was joined to generate a read count table with the unique sequences as identifier column. To remove sequences with neglectable counts, filtering was performed with Filter counttable by CPM cutoff (Galaxy Version 1.2). This resulted in a total of 51,638 and 48,833 unique sequences for EVs and embryos, respectively. These sequences were compared with NCBI BLAST + blastn (Galaxy Version 2.10.1 + galaxy1) (blastn-short) to a variety of non-coding and coding sequence collections including bovine, porcine, equine, murine, and human miRNA sequences (mature and stem-loop sequences, miRBase version 22.1), RFAM 14.7 sequences, bovine and human NCBI Refseq RNA sequences, and bovine piRNAs derived from piRBase release 2.0 (http://bigdata.ibp.ac.cn/piRBase) [56]. The annotation results were summarized to identify sequences which are annotated in miRBase as miRNAs but are probably derived from other ncRNAs. This included also the consideration of the length of the obtained sequences, i.e., if they corresponded to the known length of miRNAs (18–24 nt) [57].

### Data mining and bioinformatics analysis of RNA EVs cargo

Corresponding human miRNA identifiers were used for target gene analysis and subsequent functional annotation. To examine miRNA target genes, MIENTURNET [58] was used. Clusters of miRNAs with similar expression profiles across experimental groups were identified by the use of self-organizing tree algorithm (SOTA, Multi Experiment Viewer software v.4.8.1, https://sourceforge.net/projects/mev-tm4/) [59]. Venn diagrams were generated with Jvenn (http://jvenn.toulouse.inra.fr/app/example.html) [60] to represent miRNA comparisons among groups or miRNAs from other studies. To obtain information about overrepresented biological functions and pathways for miRNA target genes sets or clusters obtained by the different experimental groups, Metascape tool (https://metascape.org) [61] was used.

### Validation of selected miRNAs by quantitative real-time RT-PCR in EVs and embryos

Analysis of miRNA abundance for 3 selected miRNAs, based on RNA-seq results, was performed in the same EVs (17 samples: 3 replicates/group, except for EV_PE only 2 replicates) and embryos (16 samples; 4 replicates/group) by quantitative real-time RT-PCR. The miRNAs analyzed were: miR-200a, miR-184 and mir-34b-5p. Taqman miRNA assays for each selected miRNAs (#A25576, ThermoFisher Scientific, Life Technologies Europe BV, Zug, Switzerland) are listed in Table 1. First, Taqman Advanced miRNA cDNA synthesis kit (#A28007, ThermoFisher Scientific) was used to generate cDNA from total RNA of EVs and embryo samples. The same RA samples used for RNA-seq were used (0.5 ng total RNA input for embryo and 0.3 ng for EVs samples). Subsequently, miRNA abundance of the selected miRNAs was examined in the obtained cDNA samples by real time PCR on a LightCycler 96 (Roche Diagnostics (Schweiz) AG, Rotkreuz, Switzerland) with TaqMan Fast Advanced Master Mix (#4,444,556, Thermo Fisher Scientific). The real-time PCR reactions were performed in 96-well plates at a final volume of 20 μL for embryos and EVs samples. Cycle parameters of the PCR were 1 cycle of enzyme activation at 95 °C for 20 s, followed by 40 cycles of denaturation at 95 °C for 3 s, and anneal/extension at 60 °C for 30 s. A non-template control (RNA sample) was included for each primer pair. The Cq values (quantification cycle) determined for the selected miRNAs were normalized against the geometric mean of two reference miRNAs (miR-191 and miR-320). Abundancy differences between experimental groups in EV and embryo samples were calculated, and a t-test was performed in Microsoft Excel. *P*-values < 0.05 were considered significant.

**Table 1:** MicroRNA sequences and corresponding assays used for validation experiments by qPCR

**miRNAs**	**Assay reference**^**a**^	Sequence	Sequence length	miRNA differentially abundant in:
bta-miR-200a	Assay ID 478490_mir	TAACACTGTCTGGTAACGATGT	22	EVs and embryos
hsa-miR-200a-3p MIMAT0000682				
bta-miR-184	Assay ID 477938_mir	TGGACGGAGAACTGATAAGGGT	22	EVs and embryos
hsa-miR-184 MIMAT0000454				
bta-miR-34b-5p	custom assay	AGGCAGTGTAATTAGCTGATTGT	23	EVs
		bta-miR-34b-5p.1.5487		
bta-miR-191-5p	Assay ID 477952_mir	CAACGGAAUCCCAAAAGCAGCUG	23	reference miRNA
bta-miR-320a-3p	Assay ID 478594_mir	AAAAGCUGGGUUGAGAGGGCGA	22	reference miRNA

^a^All assays were purchased from: Life Technologies, Paisley, UK

### Statistical analysis

Blastocyst rates (day 7–8), flow cytometry results, EV size and EV concentrations measured by NTA are presented as mean ± SEM. All variables were previously tested for their normality (Shapiro–Wilk test). Variables following normal distribution were analyzed by a one-way analysis of variance (ANOVA) followed by the Tukey’s test for multiple comparisons. Variables not following a normal distribution were analyzed by the Kruskal–Wallis test followed by Dunn’s test for multiple comparisons. For all variables p < 0.05 was considered significant. Statistical analysis was performed by using GraphPad Prisma software, version 8.2.0 (GraphPad Software, San Diego, CA, USA) (https://www.graphpad.com/scientific-software/prism/).

## Results

### Characterization of oviductal and embryonic EVs

**Fig. 2:**
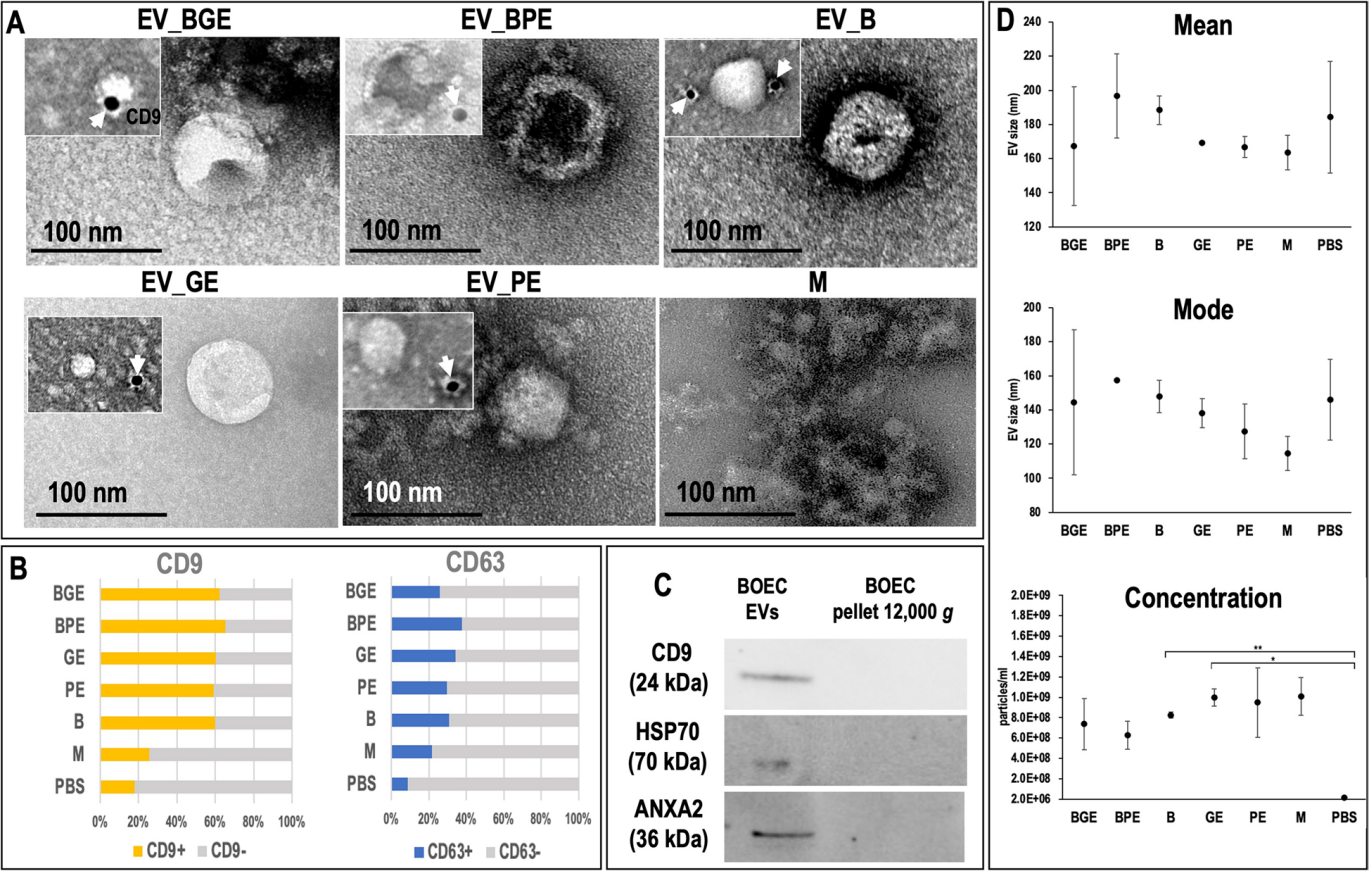
Characterization of extracellular vesicles (EVs). **A** Representative images of transmission electron microscopy (TEM) of EVs samples isolated from 6 experimental groups: EV_BGE: EVs from BOEC co-cultured with good-quality embryos; EV_BPE: EVs from BOEC co-cultured with poor-quality embryos; EV_B: EVs from BOEC cultured alone; EV_GE: EVs from good-quality embryos cultured alone; EV_PE: EVs from poor-quality embryos cultured alone; and M: medium only (control). Small images in each experimental group represent electron microphotographs of CD9 immunogold labelling of EVs. **B** Graphs representative for CD9 and CD63 expression in EVs samples, medium and PBS measured by flow cytometry. **C** Western blotting characterization of EVs and large vesicles (pellet after centrifugation of conditioned media at 12,000 g) for known EV protein markers (CD9, ANXA2 and HSP70). **D** Comparison of EVs size and concentration across samples measured by nanoparticle tracking analysis (NanoSight NS300)

Transmission electron microscopy (TEM) observations confirmed the presence of EVs in all experimental groups, except for the medium control (M) group (Fig. 2A). All samples showing the presence of EVs comprised predominantly a population of small vesicles (30–100 nm) but also showed a small population of larger vesicles (> 100 nm).

Flow cytometry results for tetraspanins CD9 and CD63 are shown in Figs. 2B and Supplementary Figure S2. The standard calibration kit containing fluorescent microspheres of known diameter (100 nm) confirmed that the CytoFLEX flow cytometer was suitable for nanoparticle measurements. Flow cytometry results demonstrated that EV populations derived from cells, embryos, or both, were positive for CD9 and CD63. The proportion of EV positive for CD9 was significantly higher (Range: 65.4 ± 7.06 – 59.2 ± 2.9%) than for CD63 (Range: 37.8.4 ± 2.8 – 25.91 ± 4.4%) in all experimental groups examined (*P* < 0.001). The M group showed values more similar to PBS (used as negative control): for CD9, M: 25.7 ± 4.7% and PBS: 18.1 ± 7.0%; and for CD63, M: 22.4 ± 0.01% and PBS: 8.8% ± 3.87%. Besides, for CD9 statistical differences (*P* < 0.05) were found in BPE vs. M, GE vs. M and PE vs. M, while for BGE vs. M (*P* = 0.051) and for B vs. M (*P* = 0.085). For CD63, statistical differences (*P* < 0.05), were found in B vs. M and GE vs. M, while BPE vs. M showed *P* = 0.08 and for PE vs. M *P* = 0.09.

Immunoblotting analysis confirmed that oEVs samples were positive for CD9, HSP70, and ANXA2 (Fig. 2C). Moreover, oEVs samples were compared to the pellet after centrifugation at 12,000 g (large vesicles and cell debris), showing the presence of bands only in oEVs compared to the large EVs (obtained from 12,000 g pellet) for the three markers (Fig. 2C). Furthermore, CD9 in EVs was also analyzed by immunogold labelling with TEM, confirming the presence of CD9-positive EVs in the samples of all experimental groups except group M (Fig. 2A).

Analysis of EVs concentration and size distribution by nanoparticle tracking analysis (NTA) did not show differences in size distribution (Fig. 2D) among experimental groups including the M group and PBS. However, when the particle concentration was analyzed, clear differences were found between all experimental groups including M group and PBS, being statistically different for B vs. PBS (*P* < 0.01) and GE vs. PBS (*P* < 0.05).

### Embryo development and BOEC viability assessments

Blastocyst rates were significantly higher for G vs. P quality embryos, regardless of BOEC co-culture for day 7 and 8 of embryo development (BGE and GE: ~ 53% vs. BPE and PE: ~ 11% at Day 8,* P* ≤ 0.001) (Table 2). BOEC viability was not affected by the co-culture with G or P quality embryos. Cell viability results were very similar among B, BGE, and BPE groups (Supplementary Figure S3) being most of the cells positive for Hoechst 33342 (in blue) and only a very few cells positive for propidium iodide (in red, dead cells).

**Table 2:** Blastocyst rates evaluated of embryos co-cultured with or without bovine oviductal epithelial cells (BOEC)

Experimental Groups	Number oocytes	% Blastocyst rate	
		**Day 7**	**Day 8**
**BGE**	330	46.92 ± 2.45^a^	54.75 ± 3.07^a^
**BPE**	230	7.83 ± 1.55^b^	9.92 ± 2.24^b^
**GE**	384	42.67 ± 2.19^a^	51.42 ± 2.78^a^
**PE**	370	7.42 ± 1.65^b^	11.50 ± 2.38^b^

Cleavage rate for all experimental groups including > 8 cells and < 8 cells at 53 h post insemination was 75.55 ± 9.23% (> 8 cells: 35.49 ± 11.97 and < 8 cells: 39.90 ± 10.03)

BGE: Good-quality embryos co-cultured with BOEC; BPE: Poor-quality embryos co-cultured with BOEC: GE: Good-quality embryos cultured without cells and PE: Poor-quality embryos cultured without cells (PE)

Data are represented as percentage of blastocyst on day 7 and day 8 of embryo development and error as standard error of the mean (SEM). Statistical comparison was performed in the same day of culture (a,b, *P* ≤ 0.001)

### Results of microRNA RNA-sequencing

**Fig. 3:**
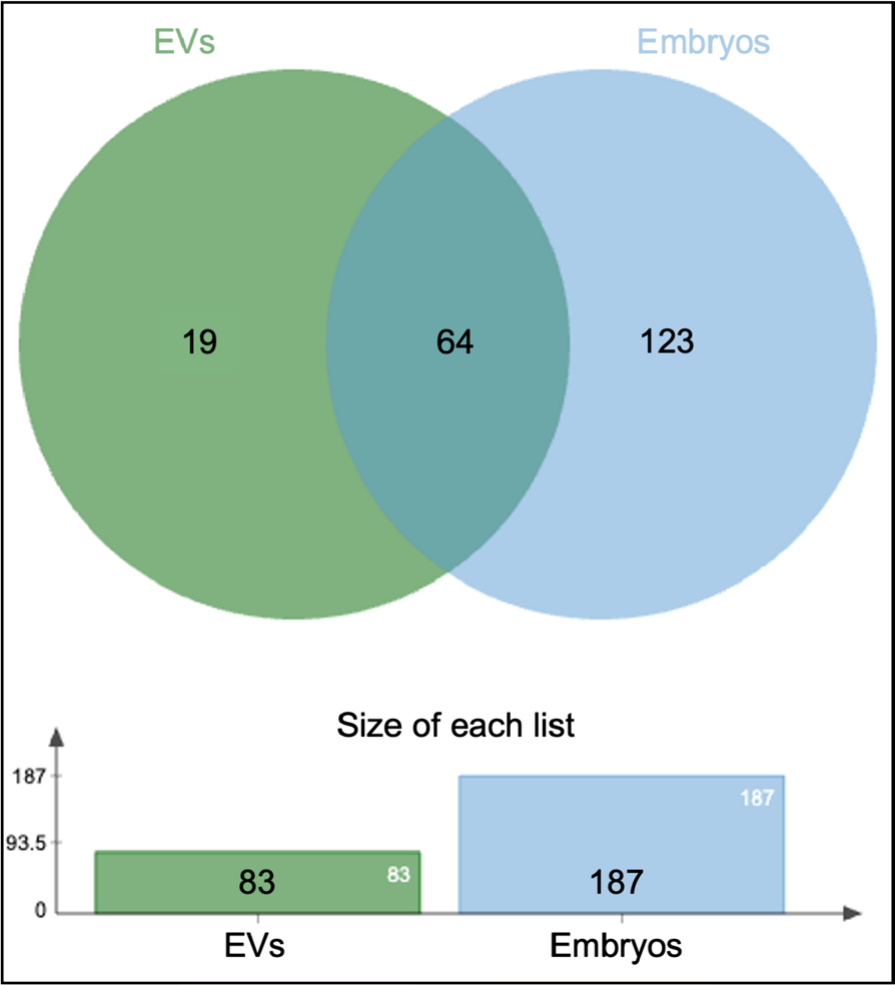
Comparison of miRNAs identified in all EVs and all embryo samples. Venn diagram comparing all miRNAs identified in EVs and in embryos. MicroRNAs only found in EVs are shown on the left (green)

From a total of 34 libraries prepared, RNA-seq analysis provided sequence data for 31 libraries, 17 EVs and 14 embryos (out of 18 and 16, respectively) (samples with no data: M_R2 and PE_R3 and GE_R3). After quality controls, two EVs samples and one embryo sample were omitted from subsequent analysis (samples removed: 2 EVs libraries EV_GE3 and EV_PE3 and 1 embryo library E_BPE). Overall, a total of 83 miRNAs were identified in EVs and 187 in embryos across samples regardless of the experimental group (Supplementary data S1-Table 1 and Supplementary data S2-Table 1). An overlap of 64 miRNAs was found between EVs and embryos, with 19 unique miRNAs in EVs and 123 in embryos (Fig. 3).

### Analysis of extracellular vesicle microRNAs

#### Differential miRNAs between oviductal and embryonic EVs

**Fig. 4:**
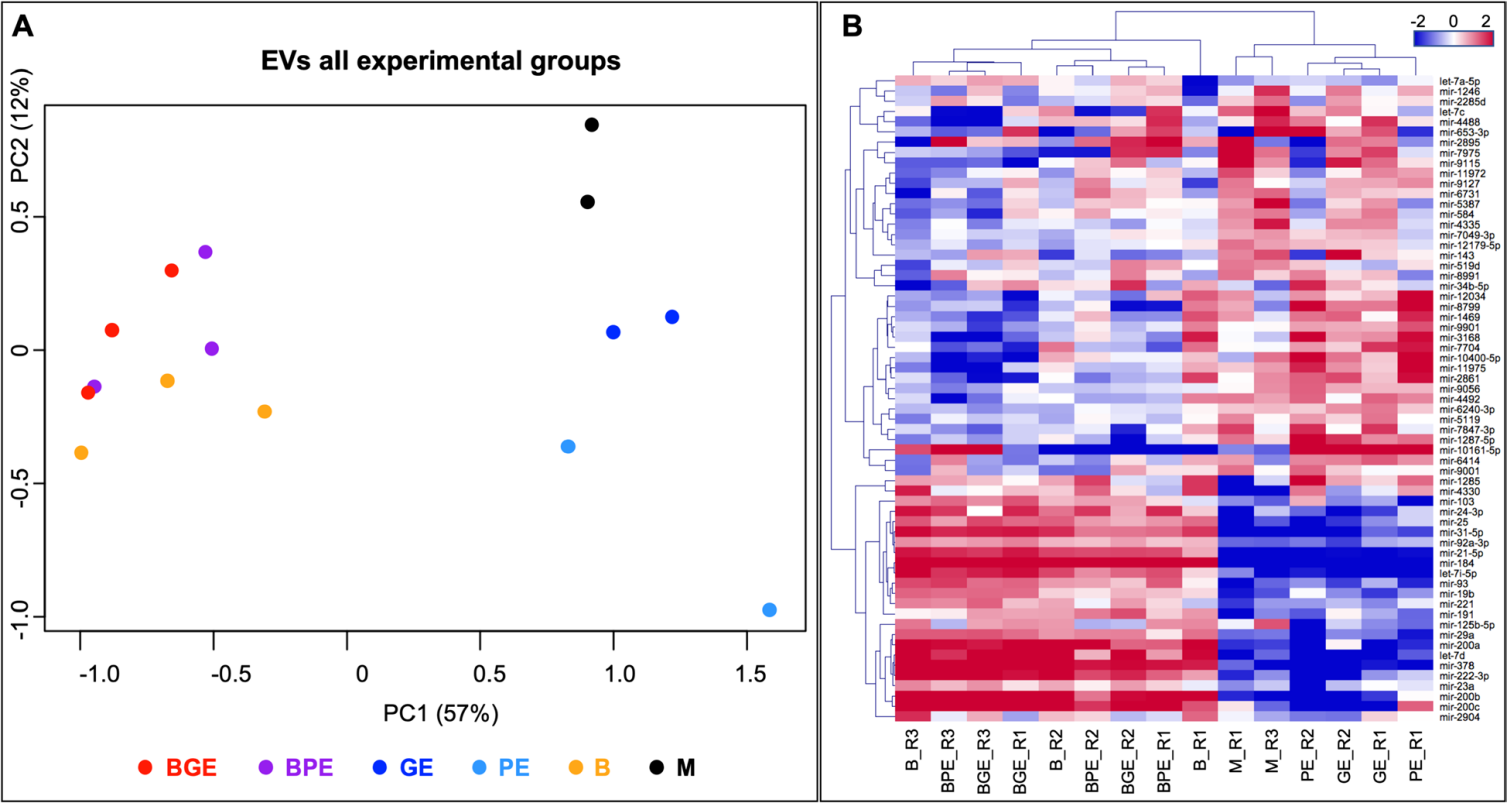
Comparative differential analysis of miRNAs in EVs across all samples represented by principal component analysis (PCA) (**A**) and unsupervised hierarchical clustering (HCL) (**B**) plots. For each HCL, rows indicate differential miRNAs, while columns represent individual EVs samples collected from the different experimental groups. Mean-centered expression values (log2 of counts per million of respective sample – mean of all samples) are shown. Color scale: blue = lower than mean, red = higher than mean. PCA and HCL images were created with Bioconductor package EdgeR (https://bioconductor.org/packages/release/bioc/html/edgeR.html) [53] and other standard R packages and modified with Adobe Photoshop v.22.4.3. Labelling of each sample refers to: EV_BGE: EVs from BOEC co-cultured with good embryo quality; EV_BPE: EVs from BOEC co-cultured with poor-quality embryo; EV_B: EVs from BOEC cultured alone; EV_GE: EVs from good embryo cultured alone; EV_PE: EVs from poor embryo cultured alone; and M: particles from media without embryos and BOEC (control). Replicates are represented by R1-R3 following the group names

Principal component analysis (PCA) (Fig. 4A) based on all identified miRNAs across EVs samples revealed a separation of EVs samples derived from BOEC-embryo co-culture (BGE and BPE) and BOEC alone (B) from EVs from embryo alone (GE and PE) and medium (M) in principal component 1. GE and PE appeared to be separated from M in principal component 2.

Unsupervised hierarchical clustering (HCL) of differentially abundant (DA) miRNAs across samples (based on *P* < 0.01 and FDR < 10%) confirmed the grouping of the samples of the co-culture groups and BOEC alone (BGE, BPE and B) and the grouping of samples from embryos alone and medium (GE, PE and M) (Fig. 4B). In addition, the two M samples separated in the sample tree from the GE and PE samples.

#### Differential EVs miRNAs between BOEC co-cultured with embryos and BOEC alone

To examine whether BOEC secrete EVs with different miRNA cargo depending on the presence or absence of embryos, the co-culture groups BGE and BPE were combined to one group (referred as BE) and compared to the BOEC alone group (B) (comparison BE vs. B). PCA based on all identified miRNAs showed that B samples separated from BE (BGE and BPE) in component 2 (Figure S4-A). This comparison resulted in 9 DA miRNAs (*P* < 0.01 and FDR < 10%, 7 out of 9 FDR < 5%), 5 with increased and 4 with decreased abundance in the presence of embryos (Table 3 and highlighted in green in Supplementary data S1-Table 2). To confirm MiRDeep2 results and considering known miRNA annotation issues [62], a subsequent small RNA annotation was performed based on BLAST comparisons to different sequence databases essentially as recently described [55]. This analysis showed that some of the DA miRNAs were annotated as probably derived from rRNA sequences or other non-coding RNAs and are marked with an asterisk in Supplementary data S1 (Table 2) and shown in Table 3.

**Table 3:** Differentially abundant miRNAs in EVs isolated from conditioned media related to embryo recognition

microRNA	log2 FC	log2 CPM	*P*-value	FDR
**BE vs. B**				
miR-2861	-1.62	9.90	0.0075	0.0691
miR-3168	-1.47	15.57	0.0000	0.0016
miR-653-3p	1.53	13.51	0.0010	0.0208
miR-2895	2.70	10.08	0.0003	0.0083
miR-7975	1.51	11.66	0.0063	0.0649
miR-8991	1.13	15.18	0.0038	0.0473
miR-10161-5p	2.28	8.89	0.0034	0.0473
**BGE vs. B**				
miR-1469	-1.71	11.98	0.0011	0.0224
miR-2861	-1.97	10.08	0.0102	0.0943
miR-3168	-1.78	15.79	0.0003	0.0109
miR-653-3p	1.71	13.47	0.0006	0.0152
miR-7975	1.41	11.33	0.0091	0.0943
miR-8991	1.29	15.17	0.0072	0.0943
**BPE vs. B**				
miR-3168	-1.33	15.91	0.0023	0.0628
miR-2895	3.11	10.11	0.0002	0.0139
miR-10161-5p	2.57	8.76	0.0031	0.0646
**BGE vs. BPE**				
miR-34b-5p	1.50	10.57	0.0301	0.9085

*FC* fold change, *CPM* counts per million, *FDR* false discovery rate

#### Differential EVs miRNAs between BOEC co-cultured with good and poor embryos

To investigate whether the oviduct secretes EVs with different miRNA cargo depending on the embryo quality, BGE vs. BPE and then, BGE vs. B and BPE vs. B were compared. Comparison between BGE and BPE provided three miRNAs with *P* < 0.05 (Table 3 and Supplementary S1 – Table 3). Comparisons between BGE vs. B and BPE vs. B miRNA profiles are illustrated in PCA plots in Figure S4-B and C. For BGE vs. B, PCA based on all identified miRNAs showed that BGE samples separated from B samples in component 2 (Figure S4-B). This comparison resulted in 8 DA miRNAs (*P* < 0.01 and FDR < 10%, 4 out of 8 FDR < 5%). Similarly, PCA for BPE vs. B showed that BPE samples separated from B samples in component 2 (Figure S4-C). This comparison resulted in 4 DA miRNAs (*P* < 0.01 and FDR < 10%, 1 out of 4 FDR < 5%). The DA miRNAs are summarized in Table 3 and shown in Supplementary S1 – Tables 4 and 5 highlighted in green.

#### Distinguishing BOEC and embryonic EVs miRNA cargo

For the comparison between EV cargo derived from BE and E, PCA based on all identified miRNAs showed that BE samples (comprising BGE and BPE) separated from E samples (comprising GE and PE) in principal component 1 (Figure S4-D). This comparison revealed 35 DA miRNAs (*P* < 0.01 and FDR < 1%) (Supplementary data S1 – Table 6). In addition, BGE vs. GE and BPE vs. PE were compared and PCA illustrating these analyses showed that both BGE and BPE separated from corresponding GE and PE samples in principal component 1 (Figure S4-E and F). These comparisons revealed 29 DA miRNAs for BGE vs. GE (*P* < 0.01, FDR < 1%) and 13 DA miRNAs for BPE vs. PE (*P* < 0.01 and FDR < 1%) (Supplementary data S1 – Tables 7 and 8).

#### Clustering of EVs miRNAs with similar expression profiles

**Fig. 5:**
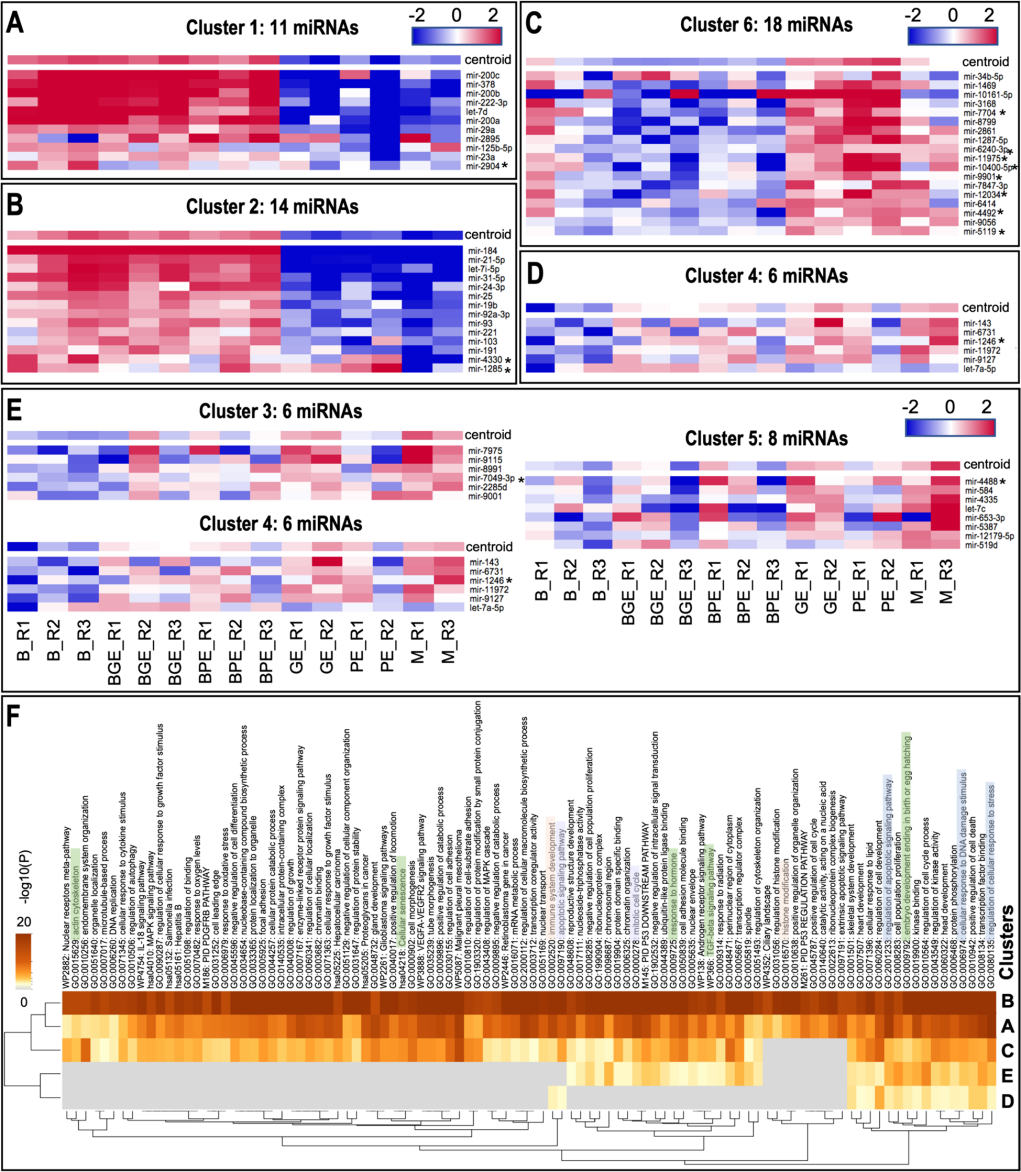
Self-organizing tree algorithm (SOTA) analysis was used to cluster the 63 differentially abundant (DA) miRNAs across EV samples with similar expression profiles by MeV. Six expression images showing the number of genes and miRNA expression profiles in each SOTA cluster are showed. The 6 miRNA clusters were further re-grouped in 5 miRNAs profiles: **A** (cluster 1), miRNAs with strong increased abundance in B, BGE, and BPE vs. BE,BP and M; **B** (cluster 2), miRNAs with a slight increased abundance in B, BGE, and BPE vs. BE,BP and M; **C** (cluster 6), miRNAs with decreased abundance in B, BGE, and BPE vs. BE, BP and M; **D** (cluster 4), miRNAs with decreased abundance only in B; and **E** (cluster 4, 5 and 3), miRNAs with increased abundance in M. **F** Functional enrichment analysis for predicted target genes of DA miRNAs grouped in A-E miRNA profiles was conducted by using Metascape tool and is illustrated in a heatmap. Bar graph of enriched terms across target genes from all differential abundant miRNAs in EVs colored by *P*-values representing enriched clusters up to a score of 2

Self-organizing tree algorithm (SOTA) was used to cluster the 63 DA miRNAs obtained from the EVs comparisons according to their expression profiles by MeV. This analysis resulted in 6 clusters with 11, 14, 6, 6, 8, and 18 miRNAs, respectively (Fig. 5, Supplementary data S1 – Table 9). The 6 miRNA clusters were further re-grouped in 5 miRNA profiles, as shown in Fig. 5A-E: profile A (cluster 1), miRNAs with highly increased abundance in B, BGE, and BPE vs. GE, PE and M; profile B (cluster 2), miRNAs with a slightly increased abundance in B, BGE, and BPE vs. GE, PE and M; profile C (cluster 6), miRNAs with decreased abundance in B, BGE, and BPE vs. GE, PE and M; profile D (cluster 4), miRNAs with decreased abundance only in B, and profile E (cluster 4, 5 and 3), miRNAs with increased abundance in M. The DA miRNAs marked with an asterisk in Fig. 5 were annotated as probably derived from rRNA sequences or other non-coding RNA on a subsequent analysis.

#### Target gene and functional annotation analysis of EVs miRNAs in clusters

Gene target analysis of the miRNAs assigned to profiles A-E was conducted by MIENTURNET (Supplementary data S1 – Table 10). This analysis resulted in 795 target genes found for profile A (2 miRNAs out of 11 found in MIENTURNET), 2865 target genes found for profile B (6 miRNAs out of 14 found in MIENTURNET), 722 target genes found for profile C (10 miRNAs out of 18 found in MIENTURNET), 44 target genes found for profile D (1 miRNA out of 6 found in MIENTURNET), and 253 target genes for E (4 miRNAs out of 20 found in MIENTURNET).

Enriched functional terms were identified for these potential target genes by using Metascape (Fig. 5F, Supplementary data S1 – Table 11). This analysis showed a high enrichment for all annotated biological functions and pathways for target genes derived from miRNAs of profile B followed by profiles A and C compared to the rest of profiles (E and D). Particularly interesting terms for these three profiles were: ‘actin cytoskeleton, ‘organelle organization’, ‘microtubule-based process’, ‘DNA replication’, ‘cellular response to cytokine stimulus’, ‘IL-18 signaling pathway’, ‘regulation of autophagy’, ‘regulation of cellular response to growth factor stimulus’, ‘response to oxygen stress’, ‘cellular senescence’, ‘cell morphogenesis ‘, ‘mRNA metabolic process ‘.

It is noteworthy to mention that target genes of miRNAs of profile C, higher amount in EVs derived from embryos and in medium controls, were not associated to some specific terms, in contrast to target genes of miRNAs of profiles A and B (miRNAs increased in EVs from B, BGE and BPE). Functional terms for the latter were, e.g., ‘ciliary landscape’, ‘histone modification’, ‘positive regulation of organelle organization’, ‘positive regulation of cell cycle’, ‘ribonucleoprotein complex biogenesis’, ‘extrinsic apoptosis signaling pathway’ and ‘PID P53 regulation pathway’. Interestingly, functional terms associated to profiles A to D and not in E (increased in media) were related to: ‘immune system development’ and ‘apoptotic signaling pathway’. Finally, it is also to be noted that some terms were found for target genes obtained for all profiles A-E, but with higher enrichment for A and B profiles, such as: ‘embryo development ending in birth or egg hatching’, ‘regulation of cell development’, ‘cellular response to DNA damage stimulus, ‘regulation of cellular response to stress’.

### Analysis of embryonic microRNAs

#### Differential embryonic miRNA profile between good and poor embryos

To compare miRNA profiles between good and poor embryos, the group comparisons GE vs. PE and BGE vs. BPE were performed. Principal component analysis based on all identified miRNAs across embryos did not reveal a separation of GE from PE samples (not shown), although 4 DA miRNAs were found (*P* < 0.01 and 2 out of 3 FDR < 5%) (Supplementary data S2 – Table 2). Principal component analysis based on all identified miRNAs across embryos co-cultured with BOEC revealed that BGE samples tend to separate from BPE samples in principal component 2 (BGE vs. BPE) (Figure S4-A). This comparison resulted in 12 DA miRNAs but was only based on two replicates per group (*P* < 0.01; 8 out of 12 FDR < 10%; 4 out of 12 FDR < 1%) (Supplementary data S2 – Table 3 and Table 4, DA miRNAs highlighted in green in supplementary). Some of these DA miRNAs were annotated in a posterior BLAST analysis as probably derived from rRNA sequences or other non-coding RNA and are marked with an asterisk and are not shown in Table 4. Besides for Table 4, only DA miRNAs with log 2 FC > 1 were selected. Finally, the 4 DA miRNAs from GE vs. PE and the 8 DA miRNAs BGE vs. BPE were compared which did not reveal an overlap.

**Table 4:** Differentially abundant miRNAs in embryos related to embryo quality and interactions with BOEC

microRNA	log2 FC	log2 CPM	*P*-value	FDR
**BGE vs. BPE**				
let-7	-2.91	9.37	0.0001	0.0001
miR-184	-1.56	8.65	0.0020	0.0523
miR-2389	-1.54	9.06	0.0016	0.0504
miR-4335	-1.61	12.33	0.0000	0.0027
miR-143	1.21	10.05	0.0071	0.1326
miR-155	1.20	11.46	0.0035	0.0817
miR-371-5p	0.86	15.89	0.0066	0.1326
miR-451	1.97	8.89	0.0001	0.0044
**GE vs. BGE**				
miR-184	-2.32	6.79	0.0004	0.0134
miR-200a	-2.05	9.30	0.0004	0.0134
miR-200b	-2.87	7.41	0.0000	0.0008
**PE vs. BPE**				
miR-184	-3.99	7.85	0.0000	0.0000
miR-200a	-2.56	9.43	0.0002	0.0052
miR-200b	-2.69	8.16	0.0002	0.0052
miR-3168	-2.08	9.39	0.0024	0.0570
miR-653-3p	3.13	12.27	0.0001	0.0036
**E vs. BE**				
miR-24-3p	-1.04	9.78	0.0003	0.0063
miR-29a	-1.21	10.55	0.0021	0.0201
miR-34a	-1.04	8.08	0.0006	0.0084
miR-141	-1.06	8.26	0.0003	0.0062
miR-184	-2.69	6.74	0.0000	0.0000
miR-200a	-2.50	9.18	0.0000	0.0000
miR-200b	-2.52	7.31	0.0000	0.0000
miR-210	-1.31	9.17	0.0026	0.0204
miR-3168	-1.70	9.42	0.0005	0.0075
miR-138	1.01	12.43	0.0002	0.0044
miR-296-3p	1.32	7.86	0.0004	0.0063
miR-301a	1.06	9.62	0.0022	0.0201
miR-653-3p	1.27	10.27	0.0094	0.0502
miR-2284x	1.18	9.83	0.0002	0.0044
miR-2370-3p	1.19	10.17	0.0006	0.0084
miR-3201	1.15	7.26	0.0028	0.0208
miR-4456	1.23	8.78	0.0004	0.0063
miR-6119-5p	1.10	9.59	0.0058	0.0370
miR-6516-3p	1.56	9.10	0.0059	0.0370

*FC* fold change, *CPM* counts per million, *FDR* false discovery rate

#### Differential miRNAs between embryos co-cultured with BOEC or cultured alone

**Fig. 6:**
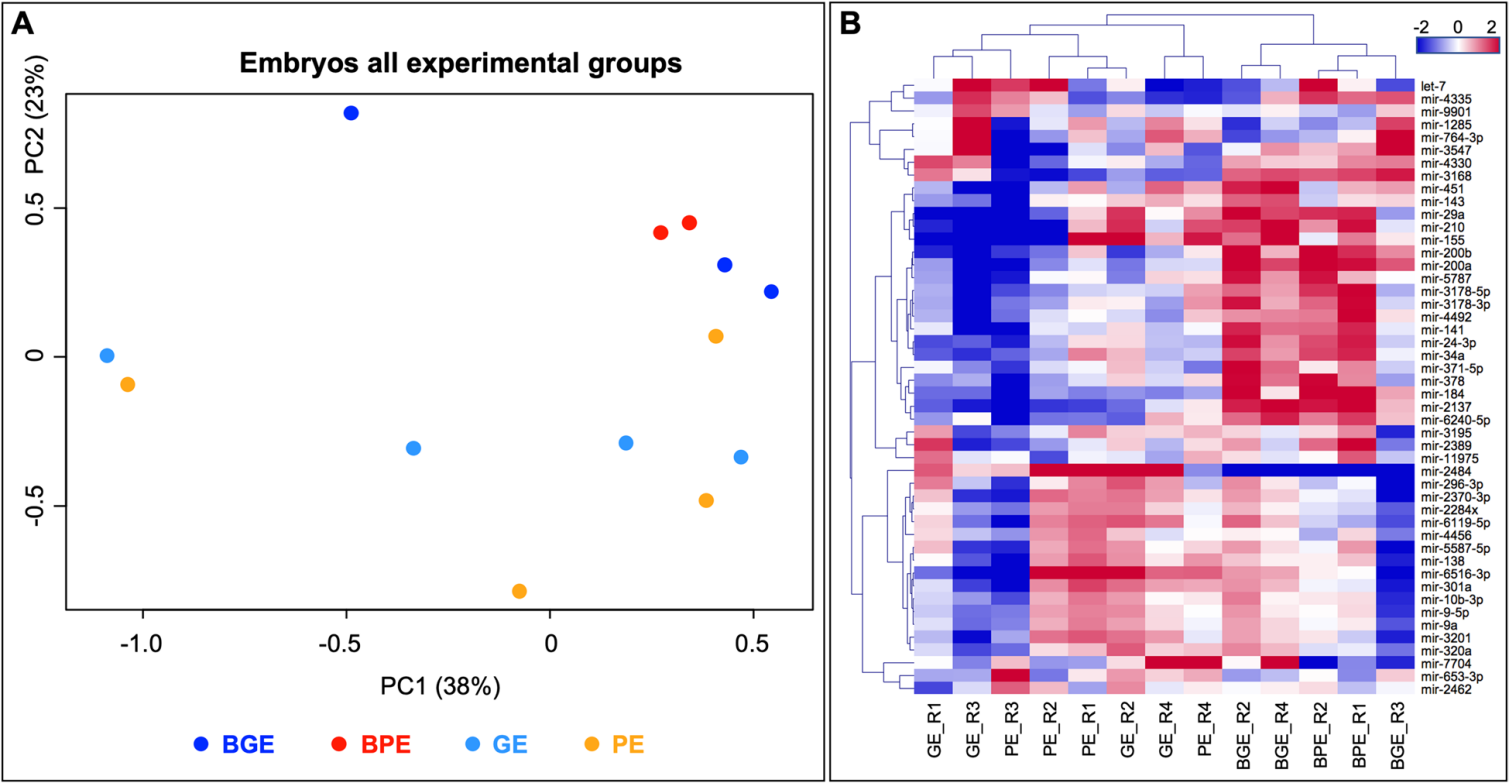
Comparative differential analysis of miRNAs in embryo across all samples represented by principal component analysis (PCA) (**A**) and unsupervised hierarchical clustering (HCL) (**B**) plots. For each HCL, rows indicate differential miRNAs, while columns represent individual embryo samples collected from different experimental groups. Mean-centered expression values (log2 of counts per million of respective sample – mean of all samples) are shown. Color scale in blue shows lower than mean and in red higher than mean. PCA and HCL images created with Bioconductor package EdgeR (https://bioconductor.org/packages/release/bioc/html/edgeR.html) 91 and other standard R packages and modified with Adobe Photoshop v.22.4.3. Labelling of each sample refers to: EB_BGE: embryo with good quality co-cultured with BOEC; EB_BPE: embryo with poor quality co-cultured with BOEC; EV_GE: embryo with good quality cultured alone; EV_PE: embryo with poor quality cultured alone; and M: media without embryos and BOEC (control). Replicates are represented by R1-R3 following the group names

First, the effect of BOEC on embryos, regardless embryo quality, was examined by comparing BE vs. E (BE = BGE + BPE; E = GE + PE). Principal component analysis based on all identified miRNAs across embryo samples revealed a separation of embryos cultured with BOEC (BE) from embryos cultured alone (E) in principal component 2 (Fig. 6A). These results were confirmed by HCL of DA miRNAs across samples (based on *P* < 0.01 and FDR < 10%), separating again embryos cultured with BOEC (BGE and BPE) from embryos cultured alone (GE and PE) (Fig. 6B). The comparison BE vs. E identified 35 DA miRNAs (*P* < 0.01 and FDR < 10%; 34 out of 35 FDR < 5%; 15 out of 35 FDR < 1%) (Supplementary data S2 – Table 4 and in Table 3 selected DA miRNAs with log2 FC > 1 and no similarities to other ncRNA sequences).

Next, the effect of BOEC on good and poor embryos was examined separately by focusing on comparisons GE vs. BGE and PE vs. BPE. Comparison GE vs. BGE resulted in 7 DA miRNAs (*P* < 0.01 and FDR < 5%), while PE vs. BPE resulted in 8 DA miRNAs (*P* < 0.01 and FDR < 10%; 5 out of 8 FDR < 5%) (Supplementary data S2 – Tables 5 and 6). Both comparations had an overlap of 5 DA miRNAs (at FDR < 10%) where 4 showed increased expression levels in embryos in co-culture with BOEC. The miRNAs miR-1285 and miR-764-3p were only DA for GE vs. BGE and miR-653-3p and miR-7704 for PE vs. BPE.

#### Clustering of embryo miRNAs with similar expression profiles

**Fig. 7:**
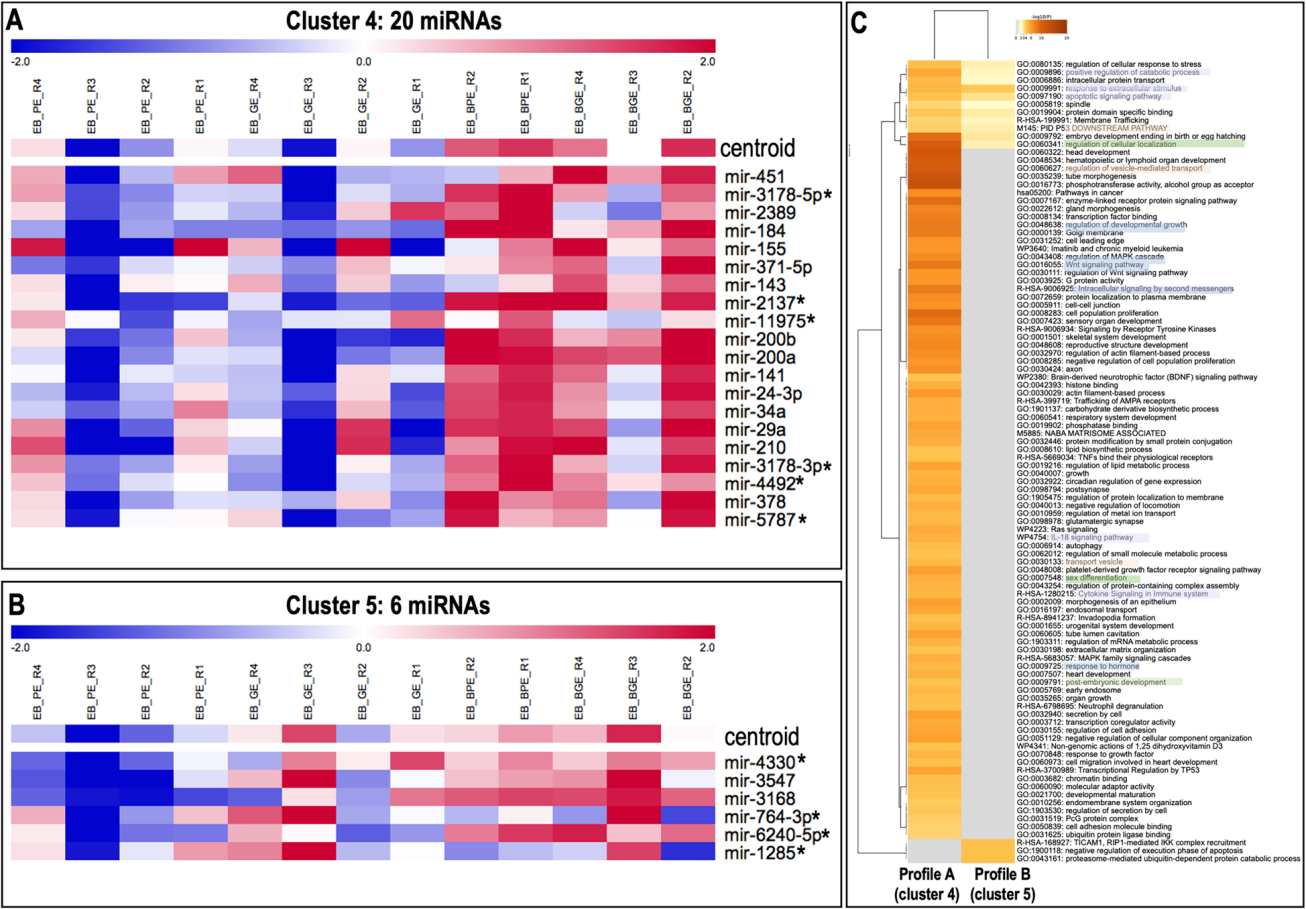
Self-organizing tree algorithm (SOTA) analysis was used to cluster the 48 differentially abundant (DA) miRNAs across embryo samples with similar expression profiles by MeV. Expression images for two of the six resulting clusters are shown with the number of miRNA expression profiles in each SOTA cluster. Two of these six miRNA clusters were further categorized as miRNAs profile A and B: **A** (cluster 4), 20 miRNAs with increase abundance in BGE and BPE vs. BE and BP; and **B** (cluster 5), 6 miRNAs with decrease abundance in BE and BP vs. BGE and BPE. **C** Functional enrichment analysis for predicted target genes of DA miRNAs grouped in A and B miRNA profiles was conducted by using Metascape tool and is illustrated in a heatmap. Bar graph of enriched terms across target genes from all differential abundant miRNAs in EVs colored by P-values representing enriched clusters up to a score of 2

Self-organizing tree algorithm (SOTA) was used to cluster the 48 DA miRNAs across embryo samples with similar expression profiles by MeV. This analysis showed that the 48 DA miRNAs were grouped in 6 clusters with 15, 1 (miR-2484/SNORD61), 2, 20, 6 and 4 miRNAs, respectively (Supplementary data S2-Table 7). Two of these 6 miRNA clusters were found interesting and were further categorized as miRNAs profiles A and B (shown in Fig. 7A). Profile A (cluster 4, 20 miRNAs) illustrated DA miRNAs with increase abundance in embryos with cells (BGE and BPE) vs. embryos alone (GE and PE). Profile B (cluster 5, 6 miRNAs), represented by DA miRNAs with decreased abundance in PE vs. GE (except for sample GE_R2), that disappeared in the presence of cells (BGE and BPE). The DA miRNAs marked with an asterisk in Fig. 7 were annotated as probably derived from rRNA sequences or other non-coding RNA on a subsequent analysis.

#### Target gene and functional annotation analysis

Target gene analysis of the miRNAs classified in A and B profiles was conducted by MIENTURNET. This analysis resulted in 782 potential target genes found for profile A (4 miRNAs out of 20 found in MIENTURNET) and 122 potential target genes found for profile B (2 miRNAs out of 6 found in MIENTURNET) (Supplementary data S2-Table 8).

Enriched functional terms were identified for these potential target genes by using Metascape (Fig. 7C and Supplementary data S2-Table 9). This analysis showed specific enrichment of biological functions and pathways for each cluster and with a small overlap among clusters. Particularly, a higher number of terms were overrepresented for A compared to B.

Only in cluster A, enriched terms were associated to, e.g., ‘regulation of vesicle-mediated transport’, ‘regulation of developmental growth’, ‘regulation of MAPK cascade’, ‘regulation of Wnt signaling pathway’, ‘intracellular signaling by second messengers’, ‘reproductive structure development’, ‘regulation of actin filament-based process’, ‘negative regulation of cell population proliferation’, ‘regulation of lipid metabolic process’, ‘Ras signaling’, ‘IL-18 signaling pathway’, ‘platelet-derived growth factor receptor signaling pathway’, ‘post-embryonic development’, ‘secretion by cell’, ‘regulation of cell adhesion’, ‘response to growth factor’ and ‘Transcriptional Regulation by TP53’. For A and B enriched terms were e.g.: ‘embryo development ending in birth or egg hatching’ (with higher enrichment in A than B profiles), ‘intracellular protein transport’, ‘regulation to cellular response to stress’, ‘response to extracellular stimulus’, ‘apoptotic signaling pathway’ and membrane trafficking’. Only in B, overrepresented terms were, e.g.: ‘TICAM1, RIP1-mediated IKK complex recruitment’, ‘negative regulation of execution phase of apoptosis’ and ‘proteasome-mediated ubiquitin-dependent protein catabolic process’.

### Comparison of EVs and embryo miRNAs with results of other studies

The 83 miRNAs identified in EVs in the present study were compared to EVs miRNA cargo derived from oviductal fluid in pregnant and non-pregnant cows [34]. With pregnant cows, an overlap of 35 miRNAs was observed (42%) while 38 miRNAs (46%) with non-pregnant cows (Supplementary data S3 – Table 1). Further comparisons with EVs from bovine oviductal fluid across the estrous cycle showed an overlap of 35 (42%) miRNAs [32] and 31 (37%) miRNAs when only the ipsilateral oviduct was considered [63] (Supplementary data S3 – Table 1). Additionally, 35 miRNAs (42%) were found in common to EVs derived from porcine oviductal fluid at the pre-ovulatory stage [30] (Supplementary data S3 – Table 1).

To examine miRNAs potentially contained in embryonic EVs, miRNAs with no read counts or very low read counts in embryos were removed from the miRNAs identified in EVs (10 out of 83 miRNAs, Supplementary data S3 – Table 2). These 73 remaining miRNAs were compared to miRNAs identified in embryonic EVs in other studies [64–68] showing an overlap of 30 (41.1%) miRNAs. Besides, these 73 miRNAs were also compared to secreted miRNAs found in the spent media of embryos [69–74], showing an overlap of 30 (41.1%) (Supplementary data S3 – Table 2).

The 187 miRNAs identified in embryos in the present study were compared to miRNAs identified in embryonic EVs in different studies (242 miRNAs) [64–68], showing an overlap of 95 miRNAs (50.8%). Besides, the 187 miRNAs were also compared to miRNAs pointed as secreted into culture media by embryos (424 miRNAs) [69–74] and resulting in an overlap of 106 miRNAs (56.7%). Finally, the 187 miRNAs identified in embryos were also compared to miRNAs identified in bovine embryos at 8 cells stage [75] (124 miRNAs), with an overlap of 71 (38%) miRNAs, respectively. The comparisons between miRNAs identified in embryos in our study and others can be found in Supplementary data S3 – Table 3.

### MicroRNA validation in EVs and embryos by quantitative real-time RT-PCR

Validation of the RNA-seq results for the selected miRNAs miR-200a, miR-184, and miR-34b-5p in embryo and EV samples was performed by real-time RT-PCR (Table 5). Real-time PCR results confirmed the RNA-seq data, although differences for miR-200a in BPE vs. PE in both embryos and EVs samples were not statistically significant but showed a tendency (embryos: *P* = 0.066 and EVs: *P* = 0.169). Besides, the slight decrease of miR-34b-5p abundance observed only in BGE vs. BPE comparison by RNA-seq (*P* = 0.030) was confirmed by a tendency to increase by PCR results (*P* = 0.0997).

**Table 5:**
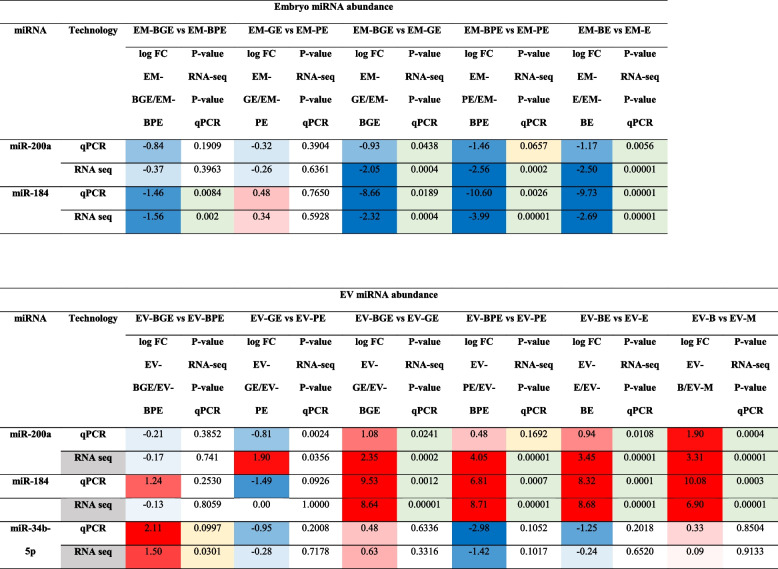
Validation of selected miRNAs in embryos and EVs samples by real-time RT-PCR

## Discussion

The findings of this study revealed novel molecular insights into the potential role of EVs in embryo-oviductal interactions. The co-culture model of oviduct epithelial cells and IVP embryos of different quality revealed differences in the miRNA cargo of secreted EVs depending on the presence and the quality of embryos. These findings suggest a potential role of oEVs in the oviduct-embryo recognition system. With respect to the other direction of embryo-oviductal interactions, the analysis of small RNAs in the embryos revealed the effects of embryo-BOEC co-culture. Despite the limitations of the in vitro model and the challenge of classifying embryos 53 h after fertilization regarding their quality (as a proxy for developmental potential), this study identified a number of miRNAs potentially involved in oviduct-embryo interactions which will be discussed the following paragraphs.

### Is the oviductal EV small RNA cargo modulated by the presence of embryos?

Both the embryo itself or the derived embryonic EVs could be recognized by the oviduct and lead to changes of the oviductal EVs RNA cargo. The comparison of EVs derived from BOEC-embryo co-culture and BOEC alone (BE vs. B; BE = BGE + BPE) revealed 9 DA miRNAs suggesting that the presence of embryos is changing EVs miRNA cargo. The comparison of these 9 DA miRNAs with miRNAs identified in EVs of pregnant cows by Mazzarella et al. [34] did not result in any overlap. However, Mazzarella et al. used a qPCR array representing 378 miRNAs derived from an older version of miRBase where most of the DA miRNAs identified here were not present. Furthermore, Mazzarella et al. compared pregnant cows after artificial insemination vs. non-pregnant cows (sham-inseminated with sperm-depleted semen extender) [34] adding the additional effect of the sperm. On the other side, limitations of our in vitro model and the period and time of embryo-BOEC co-culture could also lead to differences in the EV cargo compared to in vivo oEVs.

**Fig. 8:**
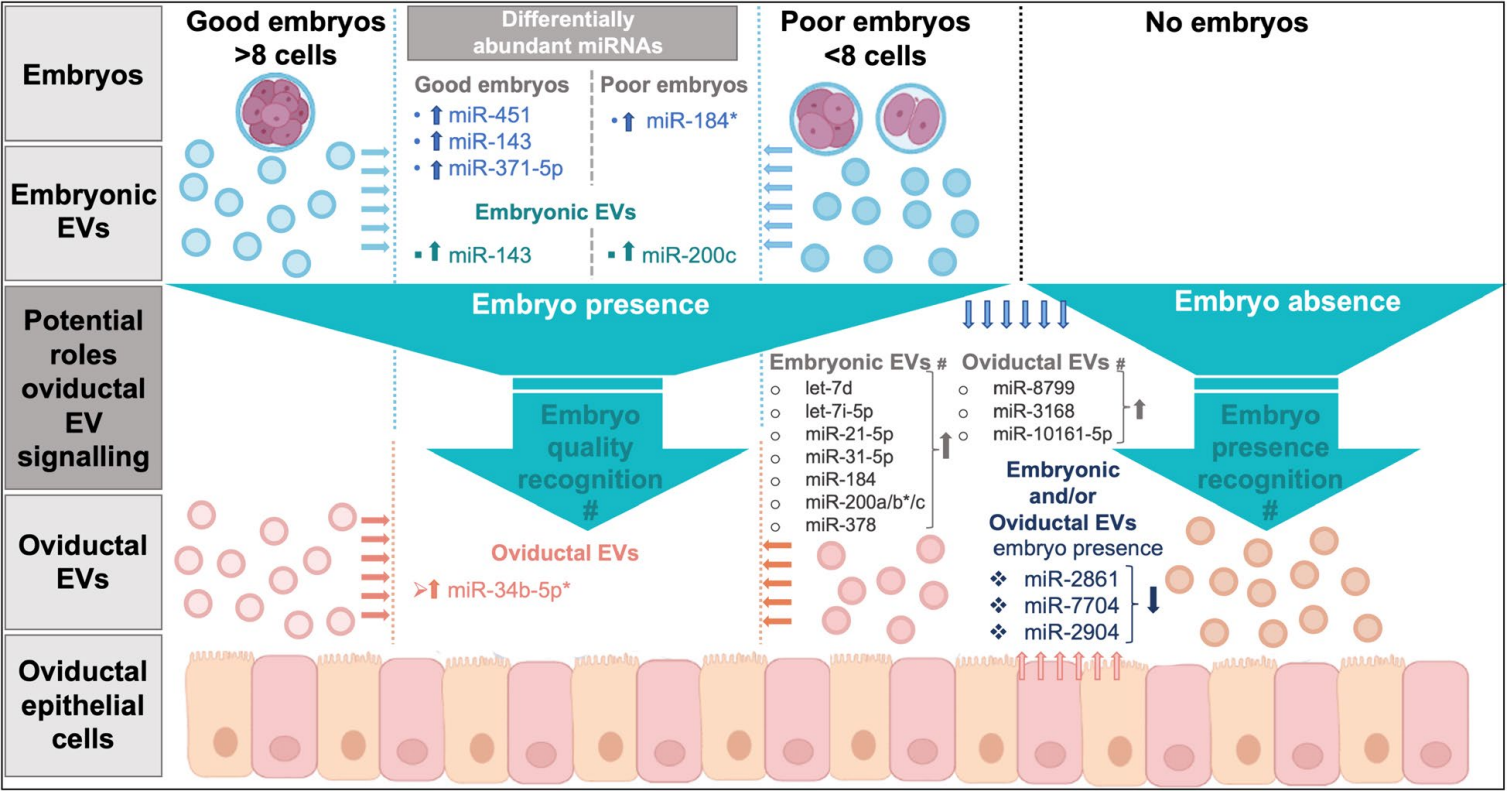
Diagram summarizing potential EV miRNAs involved in embryo recognition in the oviduct and main differences in embryonic miRNA between good and poor embryos. Embryos were classified in > 8 cells (good) or < 8 cells (poor) at 53 h post-fertilization and co-culture with or without bovine oviductal epithelial cells (BOEC) for 24 h (* miRNAs: validated by qPCR; # potential origin or potential role)

In consideration of the DA miRNAs derived from BGE vs. B (8 DA miRNAs) and BPE vs. B (4 DA miRNAs), the three miRNAs miR-7704 (DA in all three comparisons), miR-2904, and miR-2861 (DA in BE vs. B and BGE vs. B) were suggested as markers of embryo presence. Their abundance was decreased in EVs derived from embryo-BOEC co-culture (Fig. 8).

However, the detailed sequence annotation analysis showed that two of those miRNAs (miR-2904 and miR-7704) were represented with longer sequences than the usual miRNA length and had similarities to 28S rRNA sequences of the RFAM database (https://rfam.org/). These results call for caution and for further analysis with updated annotations and to confirm the potential role of these three miRNAs in the embryo oviductal recognition by functional studies.

Nevertheless, findings in the literature showed that miR-2904, miR-7704, and miR-2861 were previously identified in EVs, and support their role in early reproductive events. MiR-2904 was identified in embryonic EVs during the compaction period [66], suggesting a potential embryonic origin. Besides, miR-7704 was found increased in plasma of women with ongoing pregnancy compared to women without implantation at the timepoint of pregnancy testing [76]. MiR-2861 was among the most abundant miRNA in spermatozoa and seminal plasma of men with proven fertility [77].

### Is the oviductal EV RNA cargo different depending on embryo quality?

The comparison of EVs between BOEC-good embryo and BOEC-poor embryo co-culture (BGE vs. BPE) identified miR-34b-5p with a slight increase in BGE vs. BPE, which was further confirmed by qPCR. Therefore, the different abundance of miR-34b-5p in BGE vs. BPE could indicate a differential response of BOEC via the EVs miRNA cargo to embryo quality. This miRNA was also detected in oEVs from pregnant and non-pregnant heifers (annotated as miR-34b [34]), in oEVs from cyclic sows [30], and also in oEVs from cyclic mice [78]. In our study, miR-34b-5p was also detected in 8-cell embryos, but with no differences related to embryo quality. This suggests that EVs with differential miR-34b-5p abundance in response to G or P embryos were derived from the BOEC. Altogether, miR-34b-5p could be part of the EV RNA cargo involved in recognition of embryo quality (Fig. 8).

### Potential origin of the EVs miRNAs

Solely based on the number of oviductal and embryonic cells in the co-culture system, most of the identified miRNAs probably originated from EVs derived from BOECs. The EV comparisons co-culture vs. embryos alone (BE vs. E; 35 DA miRNAs) in consideration of the comparison of embryo EVs vs. medium control (E vs. M) confirmed this assumption. Half of the 35 DA miRNAs showed higher levels in EVs of the BE group vs. E. Among these miRNAs, let-7d, let-7i-5p, miR-21-5p, miR-31-5p, miR-184, miR-200a/b/c, and miR-378 showed at least 10-fold higher abundance in EVs derived from BE vs. E. Two of them, miR-184, miR-200b were further confirmed by qPCR. Of the miRNAs with higher abundance in EVs from embryos cultured alone, all except three (miR-3168, miR-8799, miR-10161-5p) were clearly higher in EVs from the medium only (M), showing the importance of medium controls. This finding is in line with the results of recent studies raising concerns about the effects of very difficult to remove RNA contained in common cell culture media on gene expression analysis of EVs, embryos and cells [79, 80]. Here, this could mainly affect EVs from embryos cultured alone and embryos cultured alone (high ratio of medium RNAs to RNAs in EVs or embryos).

A look at the above-mentioned three miRNAs with higher abundance in EVs from embryos on the embryo data, showed that only miR-3168 was identified in embryos but not miR-10161-5p or miR-8799. This should not discard a potential embryonic origin of these two miRNAs, since it could be possible that these two miRNAs are produced by the embryo but mainly secreted via EVs, what could explain the results obtained for EVs. Regarding miR-3168 abundance in the embryo, it was increased in embryos co-cultured with BOEC, suggesting a possible uptake of EVs secreted by BOEC by the embryo. This could explain the lower abundance in EVs derived from embryo-BOEC co-culture (BE). However, it is difficult to prove that miRNAs could be produced by the embryo but mainly secreted and therefore not possible to identify in embryos, since they are in very low amounts. Despite this limitation, our study identified several miRNAs as a result of embryo-oviduct early signaling and pointed to their likely maternal or embryonic origins (summarized in Fig. 8).

### Overlap of the obtained results with other studies

Overall, we obtained a substantial overlap between the miRNAs identified in EVs in our study with results from EVs derived from bovine and porcine oviduct (45 miRNAs, 54%). Differences in the EVs miRNA cargo could be due to the in vitro origin compared to in vivo as pointed by Almiñana et al. [27] but also due to the source of oviductal EVs from pregnant or cyclic states [32, 34] versus the different milieu of in vitro culture. As mentioned above, some of the miRNAs identified in the EVs preparations are probably derived from cell culture medium components and are probably not present in oEVs in vivo. Furthermore, differences could arise from technical and analytical differences in: 1) EV isolation; 2) miRNA analysis by RNA-seq vs. real-time PCR; 3) different small RNA library protocols, and 4) miRNA annotation. The use of different EVs isolation techniques has a significant impact on the cargo identified [50, 81]. Technical or analytical differences could also be another reason that only a bit more than half of the miRNAs identified in EVs preparations were found in these miRNA studies of in vivo oEVs. The overlap between the EVs miRNAs and miRNAs potentially contained in embryonic EVs from other studies (38 miRNAs, 46%) suggests that at least some of them could be originating from the embryo.

In contrast to the EVs results, the comparison of the embryo miRNAs with miRNAs of EVs secreted from embryos into the culture medium, miRNAs secreted from embryos into the culture medium (directly or in EVs), and miRNAs detected in 8-cell bovine embryos revealed a much higher overlap (in total 134, 72%). This is particularly supporting the obtained results in consideration that only the data set derived from Paulson et al. [75] was from day 8 bovine embryos and the present study identified a higher number of miRNAs compared to Paulson et al.

### Embryonic microRNA profiles: differences between good and poor-quality embryos

Until recently, most studies analyzed only a few selected miRNAs in 8-cell bovine embryos (miR-21-5p and miR-130a [82]; miR-10b-5p; miR-424, and miR-196a [83, 84]; miR-125a, miR-127, and miR-145 [85]), with three of them (miR-10b-5p, miR-21-5p, miR-125a) in common with our study. More recently, Paulson et al. [75] analyzed miRNAs profiles of bovine preimplantation embryos (from 1-cell to blastocyst stage) and reported embryonic miRNAs as potential regulators of embryonic transcripts, beginning at major embryonic genome activation (occurring at 8-cell stage in cattle), and later during the morula-to-blastocyst transition. The comparison of the set of embryonic miRNAs with the 124 miRNAs identified in bovine 8-cell embryos by Paulson et al. [75] showed an overlap of 71 miRNAs (57%). Interestingly, miR-371-3p was identified as a highly expressed EGA-specific miRNA in [75] and was also found in high abundance in the embryos in our study. Moreover, miR-371-5p was identified as increased in good quality compared to poor quality embryos (increased in BGE). MicroRNA gene mir-371 is a part of the mir-371–373 cluster, which is specifically expressed in human embryonic stem cells (ESCs) and functions as modulator of self-renewal and pluripotency processes, with a major regulatory role in the stemness maintenance of ESCs [86]. Besides, it plays a crucial role in preimplantation epiblast and naïve ESC [87, 88]. Altogether, it suggests an essential role of miR-371 on bovine embryo development.

Another miRNA found as increased in good vs. poor quality embryos was miR-451. Interestingly, Li et al. [89] reported that miR-451 downregulation in mouse and human oocytes affected pre-implantation embryogenesis by suppressing the Wnt signaling pathway, suggesting that miR-451 might serve as a novel biomarker of oocyte and embryo quality in ARTs. Another miRNA increased in BGE was miR-143 which has been found as involved in porcine embryo development [90]. By contrast, miR-184, with a role in supporting embryo development as mentioned above, showed lower abundance in good vs. poor embryos during co-culture with BOEC.

**Fig. 9:**
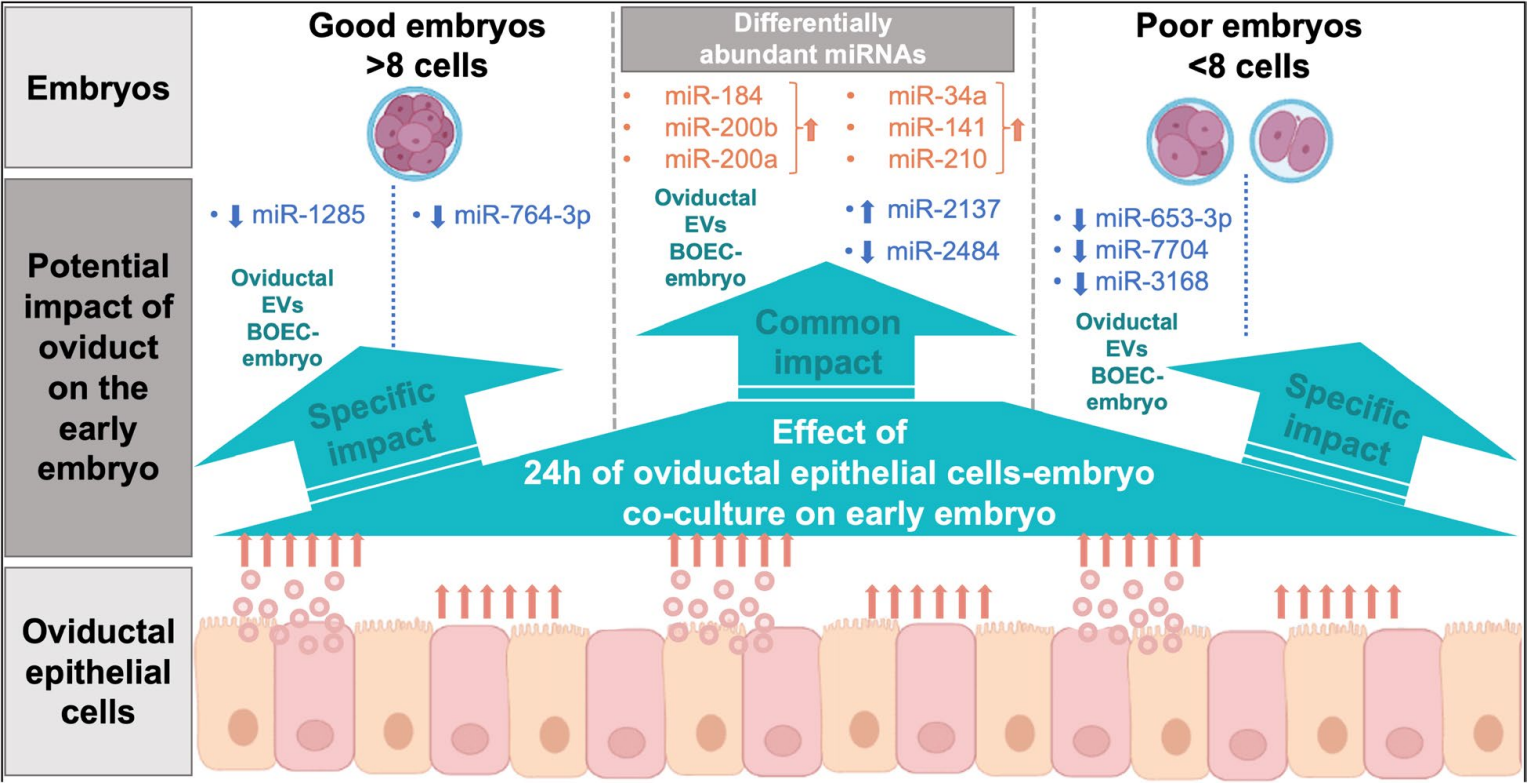
Diagram summarizing the impact of 24 h of BOEC-embryo co-culture on the miRNA profile of good and poor embryos. Embryos were classified in > 8 cells (good) or < 8 cells (poor) at 53 h post-fertilization and co-culture with or without bovine oviductal epithelial cells (BOEC) for 24 h

The functions attributed to the miRNAs DA between good and poor embryos (based on BGE and BPE comparison) indicate disturbance of developmentally important miRNAs in poor-quality embryos with retarded or arrested development. These very early differences in miRNA profiles between good and poor-quality embryos might indicate a great impact of miRNAs on the further development and fate of the embryo (DA miRNA summarized in Fig. 9).

### Impact of BOEC on early embryos: MicroRNAs differentially abundant in embryos co-cultured with BOEC or alone

Previously, Cordova et al. [91] showed that BOEC co-culture with embryos during the first four days (early stages of development) altered the transcriptome profile of blastocysts four days later, suggesting an epigenetic regulation induced by BOEC in growing embryos. Here, we showed that even only 24 h of BOEC co-culture has a clear impact on the miRNA profile of embryos at 8-cell stage (at the time of genome activation). The miRNAs discussed in this paragraph might be all potentially involved in regulation of embryo development and either taken up by the embryos from BOEC-derived EVs or were increased in embryos due to interactions with BOEC (miRNAs summarized in Fig. 9).

A number of miRNAs (miR-184, miR-200a, miR-200b) were found with higher abundance in EVs derived from BOEC-embryo co-culture vs. BOEC alone, and also in embryos co-cultured with BOEC vs. embryos cultured alone. This pattern, observed for miR-184, indicates an increased miRNA abundance in the embryo due to embryo-BOEC interactions or uptake from BOEC-derived EVs. Results from qPCR showed that miR-184 was undetectable in EVs derived from embryos as well as in embryos (detectable with very few counts by RNA-seq) but found in high abundance in EVs from BOEC co-cultured with embryos, supporting our hypothesis. MicroRNA miR-184 has been shown to be involved in multiple successive steps of oogenesis and early embryogenesis in Drosophila, including stem cell differentiation [92]. Similarly, miR-200b has been described as activated by the pluripotent stem cell activators Oct4 and Sox2, indicating a central regulatory role in ESC pluripotency [93].

In contrast to the miRNAs discussed above, miR-34a, miR-141 and miR-210 were found increased in embryos co-cultured with BOEC compared to embryos alone but were not detected in EVs samples. This suggests that they were probably upregulated in embryos due to interaction with BOEC. MicroRNA miR-34a has been described as involved in mouse ESC differentiation [94] but it also restricts development to embryonic lineages [95]. On the contrary, miR-141, as a member of the miR-200 family, was shown to maintain pluripotency of ESC [96]. In porcine embryos, miR-210 has been associated to regulation of embryo development and its repression improved cleavage and blastocyst development in IVP [97].

These results clearly demonstrate the impact of a short co-culture of BOEC (24 h) on embryos and call for rethinking strategies that can overcome the lack of early oviduct-gamete/embryo interactions in the IVP systems. In this regard, EVs have been pointed as the missing key in ARTs [19]. Potential beneficial effects of EVs as natural carriers could be achieved, e.g., by the supplementation of IVP media with EVs carrying molecules with crucial roles in oocyte maturation, sperm viability and fertilization ability, and early embryo development [21, 98]. Results from our study also point to the potential role of specific miRNAs in embryo development (miRNAs increased in good-quality embryos or in oEVs), which could be used as additives in IVP systems alone or after being loaded into EVs.

### Limitations and challenges of the experimental model compared to other studies

Our in vitro model based on BOEC monolayer and without hormonal environment could have been too simplistic compared to current 3-dimensional (3D) oviductal cell culture systems [99] and organoids [100]*.* Recent studies have shown that EVs cargo derived from 3D systems are more similar to in vivo ones than 2D cell monolayers [101]. However, at the time of the study, we did not find an established 3D model with proven embryo-oviduct interactions, allowing the embryo to develop to the blastocyst stage and easy to use in any laboratory. Moreover, 3D culture systems usually deliver lower volume of media than 2D culture systems [102], which is currently a big limitation for EVs characterization and molecular analysis of EVs cargo. In fact, the small amount of EVs and derived RNA has been one of the challenges of this study, particularly for embryos cultured alone. Moreover, the use of a combination of serial centrifugation, SEC, and ultracentrifugation to obtain EVs with higher purity (vs. higher yield) might have led to low EVs yields and very low EVs RNA input for small RNA-seq and qPCR.

Additionally, the use of 25 embryos in co-culture with BOEC in our model does not mimic the bovine physiology. However, this model has been used in different studies providing clear evidence of signaling from the embryos to the cells and vice versa [47, 91, 103, 104] and then, leading us to focus on the signaling via EVs. Moreover, it provided enough EVs and high enough amounts of RNA for downstream analysis.

The timing to assign embryos to good- or poor-quality embryo groups was based on the objectives of the study, the experimental conditions, and previous studies [42–45]. According to these studies, selecting embryos at 53 h postinsemination allows to have a pool of embryos with fast, moderate, and slow development in the “good-quality embryos” group. Selecting embryos earlier ( e.g., at 30 h) could have led to the selection of only fast embryos, which could have biased towards more male embryos. Regarding “poor-quality embryos”, developed/cleavaged embryos were selected (at 2–4-6 cells) at 53 h, with intact and good morphology of the cells. These “poor-quality” embryos could have stopped the development, or have an altered development or be only “very slow”, since part of them reached the blastocyst stage (blastocyst rate 11%), in line with previous studies [42–45]. Since more likely, there was a mix of blocked, altered but also simply delayed embryos, we did not call them “bad quality embryos” or embryos with impaired development. Thus, we used the term “poor-quality” embryos. Beyond the nomenclature and classication, the study hypothesizes that good-quality and poor-quality embryos release different signals via EVs. The different miRNA EV profiles supported our hypothesis and in somehow the classification used. A question for the next study will be, if blocked embryos or embryos with impaired development, that did not develop to blastocyst stage will signal differently than delayed embryos or embryos with an altered embryo development, that finally develop to the blastocyst stage.

Another challenging factor was the presence of precipitate particles or nanoparticles in medium samples without BOEC or embryos. The presence of these particles and associated miRNAs was a little bit surprising since a purification with SEC was performed. Similarly, Dissanayake et al. [41] reported the presence of nanoparticles in control media by NTA. Since our TEM observations clearly showed the absence of EVs in the medium control samples, the detection of particles by NTA could be due to particles derived from medium precipitates. This was further supported by flow cytometer results for CD9 and CD63 EVs markers which were similar between medium samples and PBS control. However, the miRNAs contained in the medium affect the obtained miRNA read count data. The high abundance of some miRNAs in medium samples was considered in the data analysis. In the same line, Rio and Madan [74] reported 14 different miRNAs in spent culture media without embryos [74], and Capalbo et al. [71] detected the same 11 miRNAs in spent culture media collected from individual embryos at the cleavage, the morula stages, and in the control culture medium.

Most culture systems use serum or BSA to achieve good blastocyst yield. Serum and BSA contain EVs or BSA-derived nanoparticles as previously shown [105, 106], and therefore, were not used during BOEC-embryo co-culture. This means that the precipitate and miRNAs found in medium control samples might come from the medium itself. Our results and others [79, 107] suggest that commercial media formulations or homemade prepared media used for IVP might contain miRNAs (derived or not derived from EVs), which can affect embryo culture and miRNA analysis. These results call for attention, since many published results could be biased by these medium miRNAs and the medium-contained RNAs could have adverse effects on embryo development and even on the future offspring.

## Conclusions

The findings of the present study revealed a specific response of oviductal epithelial cells towards the presence of embryos in terms of changes in the miRNA cargo of secreted EVs. This oviductal EV response varied depending on the embryo quality. These results were supported by the different miRNA profile between good- and poor-quality embryos (8-cell stage). Furthermore, embryo miRNA datasets revealed the big impact that only 24 h of co-culture with oviductal epithelial cells can exert on the early embryo, which varied also depending on the embryo quality. The integrative miRNA analysis of oviductal EVs and embryos points to specific miRNAs, which might be key in supporting an appropriate oviductal-embryo signaling as well as embryo development and growth. Moreover, it allowed to suggest the oviductal or embryonic EVs origin for those miRNAs. Altogether, our findings point to oviductal EVs as a part of the oviductal recognition system of the presence and quality of the embryo.

### Supplementary Information

Supplementary Material 1: Supplementary data S1. MicroRNA profiling of EVs. Table 1. Overview of statistical comparison results and expression profiles of all miRNAs identified in EVs across all samples. Table 2. MicroRNAs with differential abundance (DA) in EV collected from BOEC co-cultured with embryos versus BOEC cultured (BE vs. B). Table 3. DA miRNAs in EV collected from BOEC co-cultured with good embryos versus BOEC co-cultured with poor embryos (BGE vs. BPE). Table 4. DA miRNAs in EV collected from BOEC co-cultured with good embryos versus BOEC cultured (BGE vs. B). Table 5. DA miRNAs in EV collected from BOEC co-cultured with poor embryos versus BOEC cultured (BPE vs. B). Table 6. DA miRNAs in EV collected from BOEC co-cultured with embryos versus embryos cultured alone (BE vs. E). Table 7. DA miRNAs in EV collected from BOEC co-cultured with good embryos versus good embryos cultured alone (BGE vs. GE). Table 8. DA miRNAs in EV collected from BOEC co-cultured with poor embryos versus poor embryos cultured alone (BPE vs. PE). Table 9. All DA miRNAs across EV samples used for SOTA analysis and list of miRNAs in the 6 clusters. Table 10. List of predicted target genes for miRNAs classified in A-E SOTA profiles identified by MIENTURNET tool. Table 11. Functional annotation enrichment analysis for predicted target genes for miRNAs classified in A-E SOTA expression profiles across EV samples conducted by Metascape. Table 12. DA miRNAs in EV collected from BOEC cultured alone versus medium controls (B vs. M). Table 13. DA miRNAs in EV collected from good embryos versus poor embryos cultured alone (GE vs. PE). Table 14. DA miRNAs in EV collected from embryos cultured alone (considering GE and PE together) versus medium (E vs. M).

Supplementary Material 2: Supplementary data S2. MicroRNA profiling of embryos. Table 1. Overview of statistical comparison results and expression profiles of all miRNAs identified in embryos across all samples. Table 2. DA miRNAs in good embryos cultured alone versus poor embryos cultured alone (GE vs. PE). Table 3. DA miRNAs in good embryos co-cultured with BOEC versus poor embryos co-cultured with BOEC (BGE vs. BPE). Table 4. DA miRNAs in embryos co-cultured with BOEC (considering BGE and BPE together) versus embryos cultured alone (considering GE and PE together) (BE vs. E). Table 5. DA miRNAs in good embryos cultured alone vs. good embryos co-cultured with BOEC (GE vs. BGE). Table 6. DA miRNAs in poor embryos cultured alone vs. poor embryos co-cultured with BOEC (PE vs. BPE). Table 7. All DA miRNAs across embryo samples used for SOTA analysis and list of miRNAs in the 6 clusters of similar expression profiles. Table 8. List of predicted target genes for miRNAs classified in SOTA profiles A and B across embryo samples identified by MIENTURNET tool. Table 9. Functional enrichment analysis and annotation for predicted target genes for miRNAs of A and B SOTA expression profiles across embryo samples conducted by Metascape.

Supplementary Material 3: Supplementary data S3. Integrative analysis of EV and embryos datasets and comparative analysis with other studies. Table 1. Comparison of EV miRNAs identified in in our study to oviductal EV miRNAs from other studies. Table 2. Comparison of EV miRNAs identified in in our study to embryonic EV miRNAs from other studies. Table 3. Comparison of miRNAs identified in embryos in our study to other studies. Table 4. Lists of target genes of miR-184 and miR-200a obtained by using MIENTURNET. Table 5. Functional enrichment analysis of target genes of miR-184 by using Metascape. Table 6. Functional enrichment analysis of target genes of miR-200a by using Metascape.

Supplementary Material 4: Supplementary Figure S1. Flow Cytometry controls for EV characterization. (A) Plot representing flow cytometry analysis of a mix of polystyrene nanobeads from different sizes. A gate was established to detect EVs based on their size (nanoparticles with a diameter between 100 to 300 nm) to distinguish true events from electronic noise and increase the specificity of EVs detection events in the EV gate. (B) Representative plots of PBS plus antibodies used as negative control.

Supplementary Material 5: Supplementary Figure S2. Characterization of extracellular vesicles (EVs) from the different experimental groups by flow cytometry for known EV membrane markers. For each EV marker, positive (EVs isolated from oviductal fluid) and negative (PBS, in green) controls were used. Representative graphs of CD63 and CD9 expression in EVs samples measured by flow cytometry are shown.

Supplementary Material 6: Supplementary Figure S3. Evaluation of bovine oviductal epithelial cells (BOEC) viability after co-culture with good embryos (GE), poor embryos (PE) or alone. BOEC were stained with Hoechst 33342 (blue; all cells) and propidium iodide; (red; dead cells) and observed by fluorescence microscopy. No differences in viability were observed between BOEC co-culture with poor or good embryo or alone.

Supplementary Material 7: Supplementary Figure S4. Principal component analysis (PCA) based on miRNA for EV collected from co-culture of BOEC with good and poor embryo versus BOEC alone and versus embryos alone (A) PCA representing comparison BE vs. B: BOEC co-cultured with good embryo quality (EV_BGE) and poor embryo quality (EV_BPE) versus EVs from BOEC alone (EV_B). (B) PCA representing comparison BGE vs. B: BOEC co-cultured with good embryo quality (EV_BGE) versus EVs from BOEC alone (EV_B). (C) PCA representing comparison BPE vs. B: BOEC co-cultured with poor embryo quality (EV_BGE) versus EVs from BOEC alone (EV_B). (D) PCA representing comparison BE vs. E: BOEC co-cultured with good embryo quality (EV_BGE) and poor embryo quality (EV_BPE) versus EVs from good and poor embryo quality cultured alone (EV_GE and EV_PE). (E) PCA representing comparison BGE vs. E: BOEC co-cultured with good embryo quality (EV_BGE) versus EVs from good embryos cultured alone (EV_GE). (F) PCA representing comparison BPE vs. E: BOEC co-cultured with poor embryo quality (EV_BPE) versus EVs from poor embryos cultured alone (EV_PE). Replicates of each experimental group are represented by R1-R3 following the group names.

Supplementary Material 8: Supplementary Figure S5. Principal component analysis (PCA) based on miRNA for embryos with different quality co-culture of BOEC or alone. (A) PCA representing comparison BGE vs. BPE: good quality embryo co-cultured with BOEC (EB_BGE) versus poor quality embryo co-cultured with BOEC (EB_BPE). (B) PCA representing comparison BGE vs. GE: good quality embryo co-cultured with BOEC (EB_BGE) versus good quality embryo cultured alone (EB_GE). (C) PCA representing comparison BPE vs. PE: poor quality embryo co-cultured with BOEC (EB_BPE) versus poor quality embryo cultured alone (EB_GE). Replicates of each experimental group are represented by R1-R3 following the group names.

Supplementary Material 9.

## Data Availability

RNA-Seq data have been deposited at NCBI’s Sequence Read Archive (SRA), BioProject accession number PRJNA928588 (http://www.ncbi.nlm.nih.gov/bioproject/928588). Further data supporting the findings of this study are available from the corresponding author upon reasonable request.
