# Supplementary figures and images for: Oviductal extracellular vesicles miRNA cargo varies in response to embryos and their quality
Source: BMC Genomics. 2024 May 27;25:520. doi: 10.1186/s12864-024-10429-5 (PMC11129498; doi:10.1186/s12864-024-10429-5)

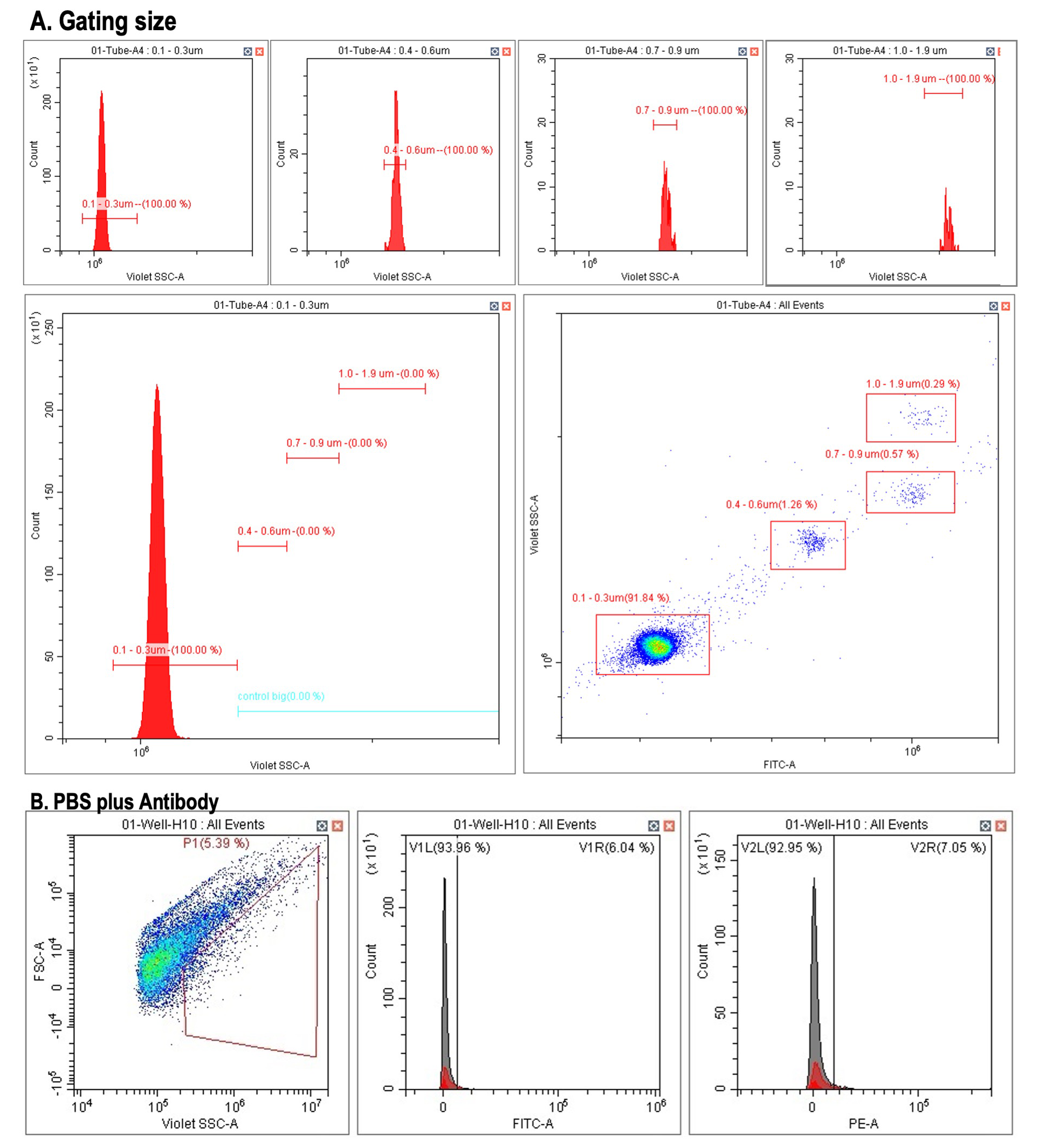

Supplement: Supplementary file 4 — Supplementary Material 4: Supplementary Figure S1. Flow Cytometry controls for EV characterization. (A) Plot representing flow cytometry analysis of a mix of polystyrene nanobeads from different sizes. A gate was established to detect EVs based on their size (nanoparticles with a diameter between 100 to 300 nm) to distinguish true events from electronic noise and increase the specificity of EVs detection events in the EV gate. (B) Representative plots of PBS plus antibodies used as negative control. [file 12864_2024_10429_MOESM4_ESM.tif]

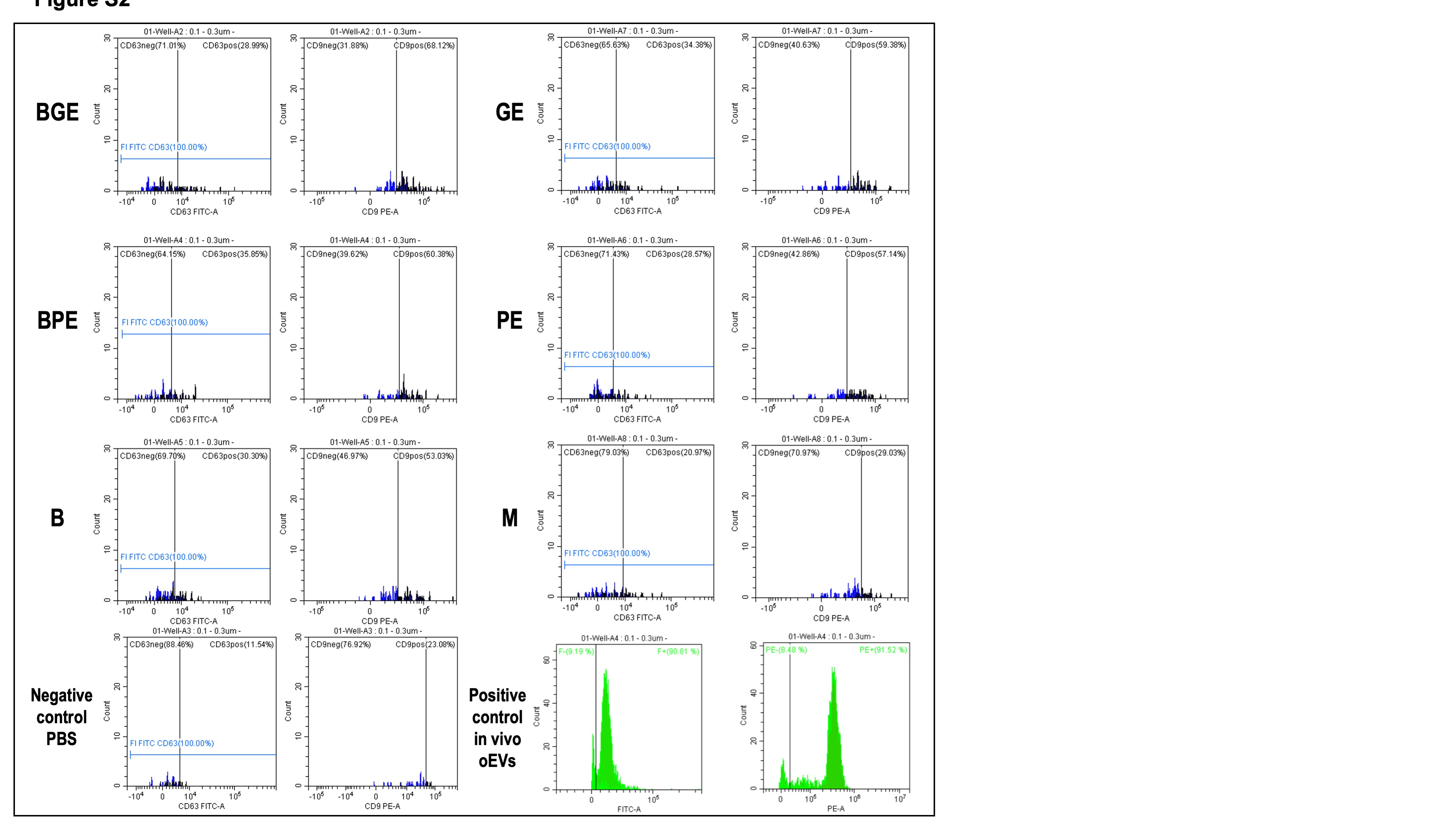

Supplement: Supplementary file 5 — Supplementary Material 5: Supplementary Figure S2. Characterization of extracellular vesicles (EVs) from the different experimental groups by flow cytometry for known EV membrane markers. For each EV marker, positive (EVs isolated from oviductal fluid) and negative (PBS, in green) controls were used. Representative graphs of CD63 and CD9 expression in EVs samples measured by flow cytometry are shown. [file 12864_2024_10429_MOESM5_ESM.jpg]

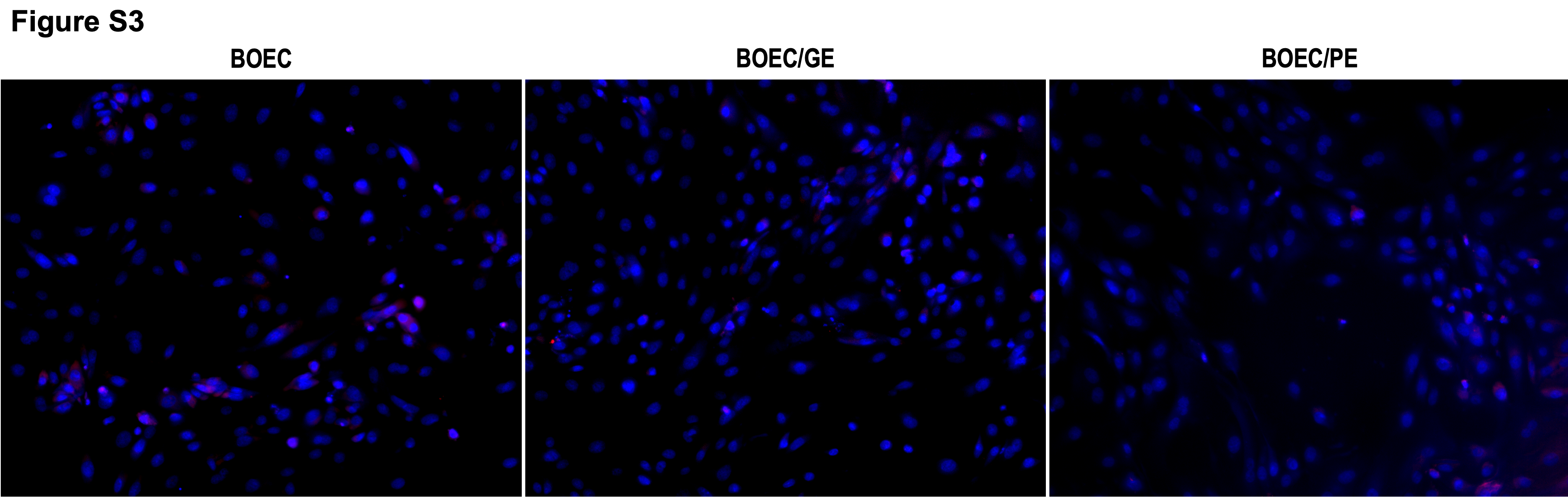

Supplement: Supplementary file 6 — Supplementary Material 6: Supplementary Figure S3. Evaluation of bovine oviductal epithelial cells (BOEC) viability after co-culture with good embryos (GE), poor embryos (PE) or alone. BOEC were stained with Hoechst 33342 (blue; all cells) and propidium iodide; (red; dead cells) and observed by fluorescence microscopy. No differences in viability were observed between BOEC co-culture with poor or good embryo or alone. [file 12864_2024_10429_MOESM6_ESM.tif]

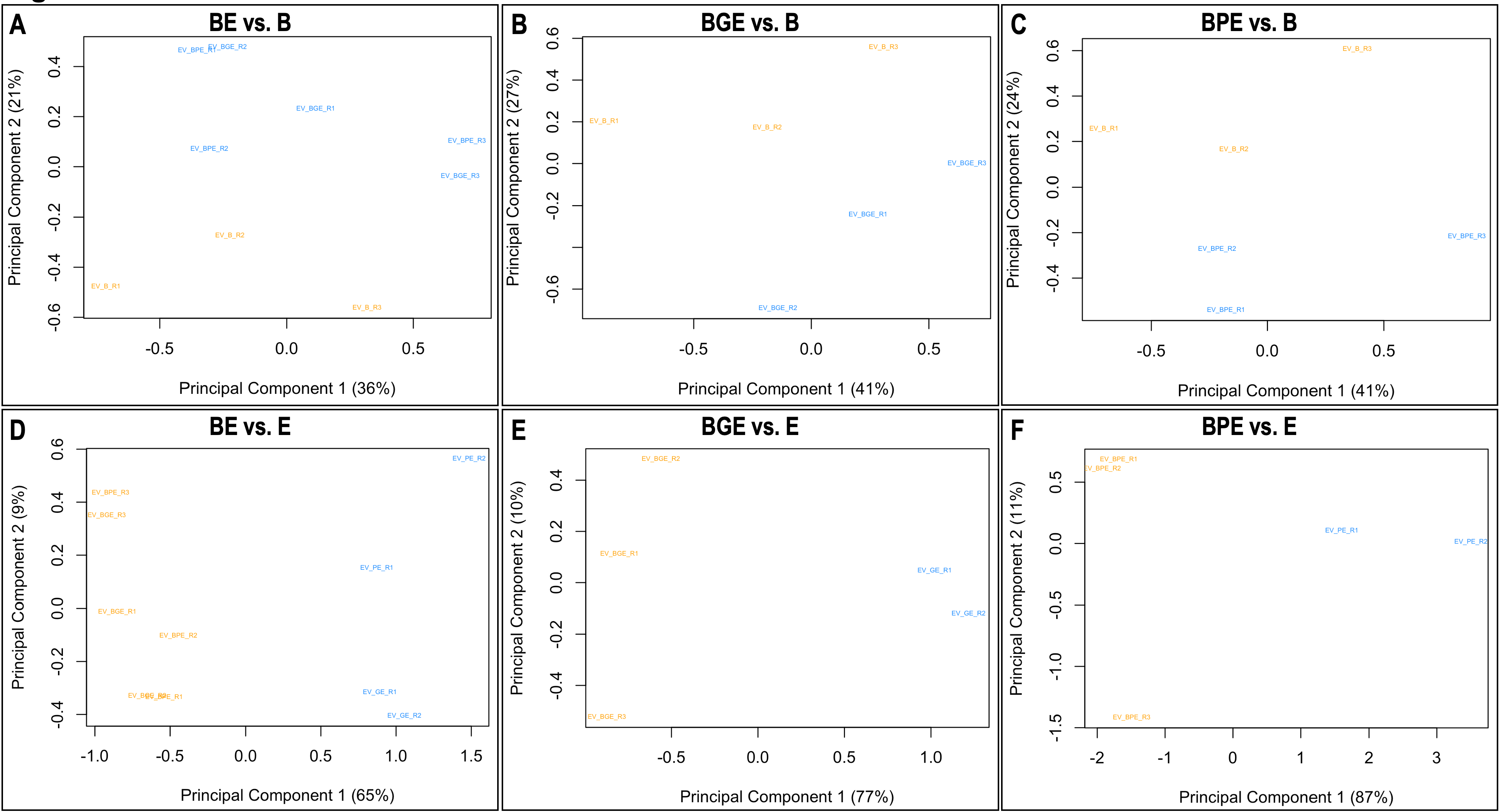

Supplement: Supplementary file 7 — Supplementary Material 7: Supplementary Figure S4. Principal component analysis (PCA) based on miRNA for EV collected from co-culture of BOEC with good and poor embryo versus BOEC alone and versus embryos alone (A) PCA representing comparison BE vs. B: BOEC co-cultured with good embryo quality (EV_BGE) and poor embryo quality (EV_BPE) versus EVs from BOEC alone (EV_B). (B) PCA representing comparison BGE vs. B: BOEC co-cultured with good embryo quality (EV_BGE) versus EVs from BOEC alone (EV_B). (C) PCA representing comparison BPE vs. B: BOEC co-cultured with poor embryo quality (EV_BGE) versus EVs from BOEC alone (EV_B). (D) PCA representing comparison BE vs. E: BOEC co-cultured with good embryo quality (EV_BGE) and poor embryo quality (EV_BPE) versus EVs from good and poor embryo quality cultured alone (EV_GE and EV_PE). (E) PCA representing comparison BGE vs. E: BOEC co-cultured with good embryo quality (EV_BGE) versus EVs from good embryos cultured alone (EV_GE). (F) PCA representing comparison BPE vs. E: BOEC co-cultured with poor embryo quality (EV_BPE) versus EVs from poor embryos cultured alone (EV_PE). Replicates of each experimental group are represented by R1-R3 following the group names. [file 12864_2024_10429_MOESM7_ESM.tif]

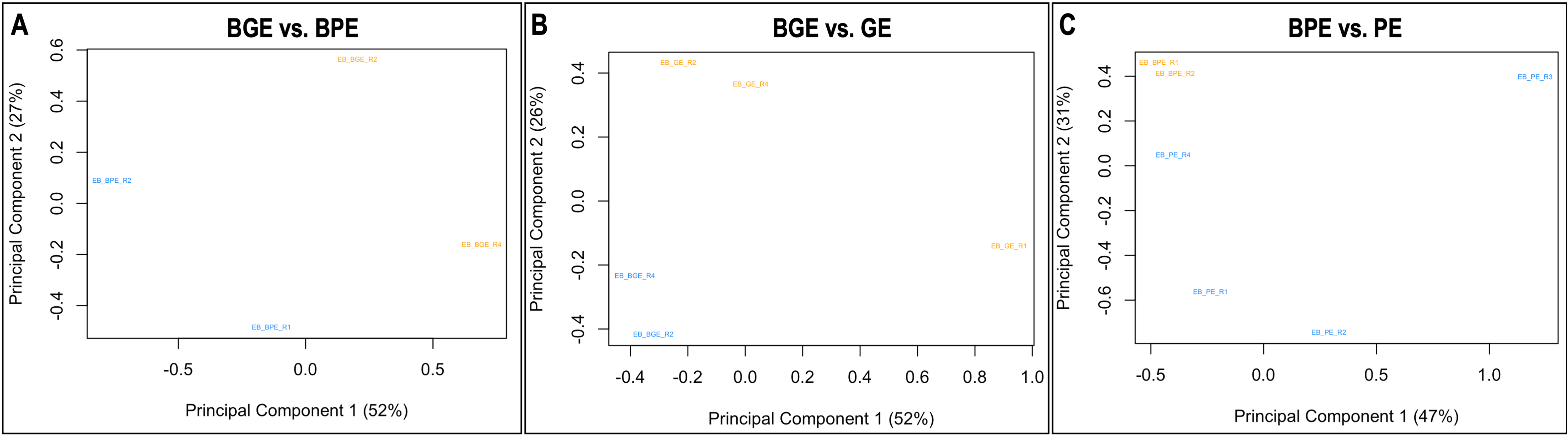

Supplement: Supplementary file 8 — Supplementary Material 8: Supplementary Figure S5. Principal component analysis (PCA) based on miRNA for embryos with different quality co-culture of BOEC or alone. (A) PCA representing comparison BGE vs. BPE: good quality embryo co-cultured with BOEC (EB_BGE) versus poor quality embryo co-cultured with BOEC (EB_BPE). (B) PCA representing comparison BGE vs. GE: good quality embryo co-cultured with BOEC (EB_BGE) versus good quality embryo cultured alone (EB_GE). (C) PCA representing comparison BPE vs. PE: poor quality embryo co-cultured with BOEC (EB_BPE) versus poor quality embryo cultured alone (EB_GE). Replicates of each experimental group are represented by R1-R3 following the group names. [file 12864_2024_10429_MOESM8_ESM.tif]

## Supplementary information Western Blotting

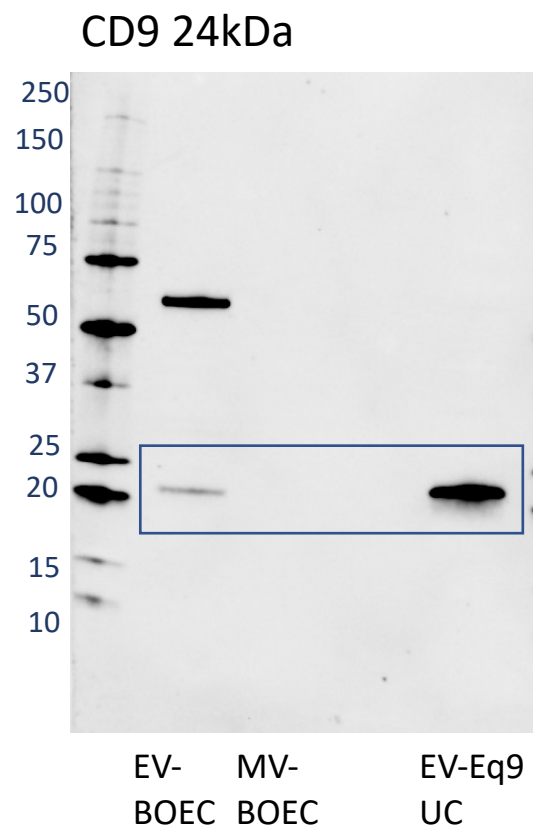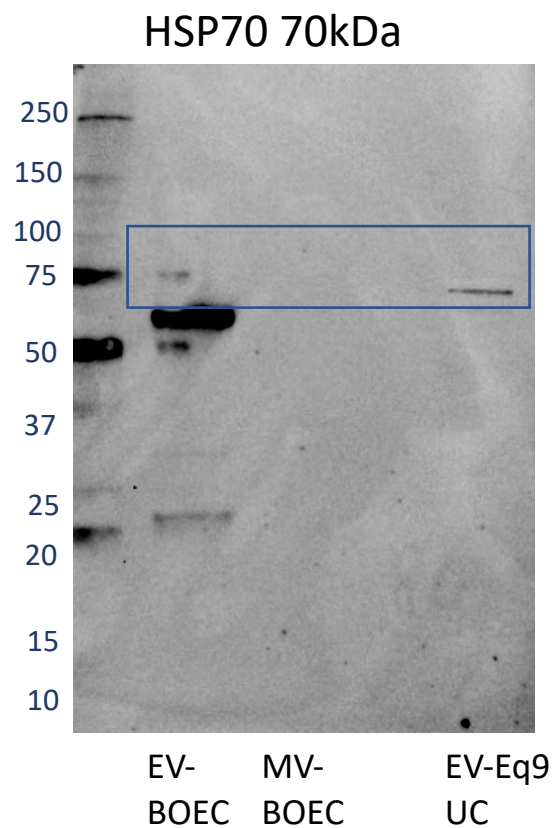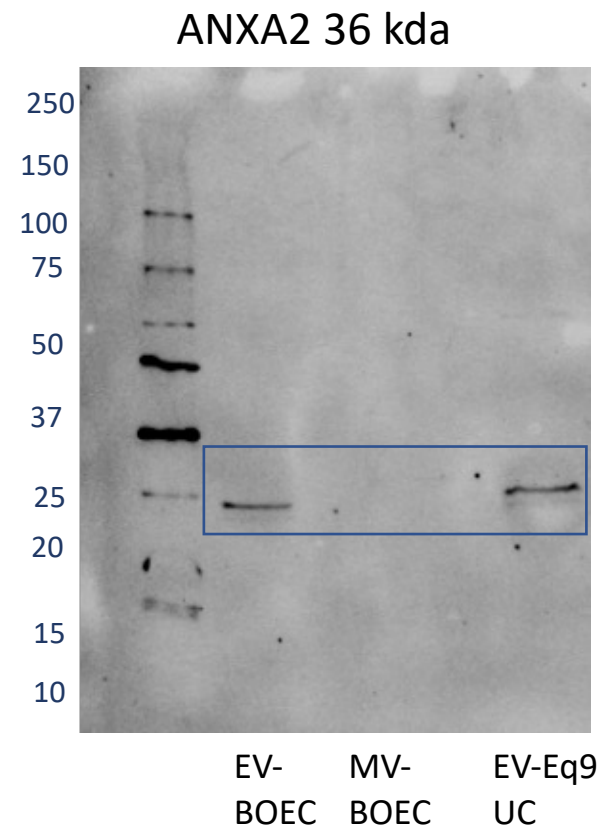

Supplement: Supplementary file 9 — Supplementary Material 9. [file 12864_2024_10429_MOESM9_ESM.pdf]
